# Transition Metal Carbides and Nitrides in Energy Storage and Conversion

**DOI:** 10.1002/advs.201500286

**Published:** 2016-02-04

**Authors:** Yu Zhong, Xinhui Xia, Fan Shi, Jiye Zhan, Jiangping Tu, Hong Jin Fan

**Affiliations:** ^1^ State Key Laboratory of Silicon MaterialsKey Laboratory of Advanced Materials and Applications for Batteries of Zhejiang Province, and Department of Materials Science and EngineeringZhejiang UniversityHangzhou310027P. R. China; ^2^ School of Physical and Mathematical SciencesNanyang Technological UniversitySingapore637371Singapore

**Keywords:** electrocatalyst, energy storage, metal carbides, metal nitrides, supercapacitors

## Abstract

High‐performance electrode materials are the key to advances in the areas of energy conversion and storage (e.g., fuel cells and batteries). In this Review, recent progress in the synthesis and electrochemical application of transition metal carbides (TMCs) and nitrides (TMNs) for energy storage and conversion is summarized. Their electrochemical properties in Li‐ion and Na‐ion batteries as well as in supercapacitors, and electrocatalytic reactions (oxygen evolution and reduction reactions, and hydrogen evolution reaction) are discussed in association with their crystal structure/morphology/composition. Advantages and benefits of nanostructuring (e.g., 2D MXenes) are highlighted. Prospects of future research trends in rational design of high‐performance TMCs and TMNs electrodes are provided at the end.

## 1 Introduction

To maintain the economic growth of modern society and simultaneously suitability of the Earth, it is urgent to search new and clean energy sources, and also improve the utilization efficiency of the primary energy sources.^1^, ^2^ All the clean energy obtained from nature, such as solar, tidal, geothermal, and wind powers, need be converted from currents to other forms for storage and resupply, mainly electrochemical energies. Hence, high‐efficiency intermediate devices for energy storage and conversion are indispensable. To date, there are two main types of devices dominating in the area of energy storage and conversion. One is fuel cell, the other is electrochemical energy storage (EES) devices including various types of batteries and supercapacitors (SCs). The fuel cell can generate electricity arising from the electrochemical reactions between hydrogen and oxygen, which are coupled with water splitting technology driven by solar energy. Fuel cell is considered as the main futuristic power source due to its high performance and infinitely renewable characteristics, but the overwhelming roadblocks related to high‐cost and unreliability of Pt‐based electrocatalysts make it impractical for large‐scale manufacture at this stage. The electrode for fuel cells consists of active electrocatalysts and support matrix, and the active electrocatalyst is the key factor to the performance of fuel cells. Current trend has turned to nonprecious high‐performance electrocatalysts instead of noble metals to drive the commercial application.^3^, ^4^, ^5^ Meanwhile, in parallel with the research of fuel cell, great efforts are dedicated to developing new‐generation EES technologies to meet the increasing demand in both consumable electronics and electric transport systems. Current dominating EES systems include Li ions batteries (LIBs),^6^ sodium ion batteries (SIBs),^7^ and SCs,^8^ with high energy and power densities^9^ as well as long cycling stability.^10^ It is a consensus that the future of EES devices depends mainly on the development/breakthrough of advanced active electrode materials.

Transition metal carbides (TMCs) and transition metal nitrides (TMNs) have been interesting active materials for electrodes owing to their advantageous physical properties, among which is their high melting points (e.g., tantalum carbide and hafnium carbide have among the highest known melting points of all materials). Moreover, they display high electrical conductivity and chemical stability as well. More detailed information on the associated fundamental properties are listed in **Table** **1**. TMCs and TMNs were introduced in the mid‐21st century and followed with continuous discovery of its distinguishing mechanical and chemical properties, which opened the door for the commercial applications of TMCs and TMNs as cutting tools, rotors within gas turbines, and as protective coatings within fusion reactors.^11^ The discovery in Pt‐like catalytic behavior of tungsten carbide (WC) in 1973 (Levy and Boudart's report^12^ has advanced the progress of their potential electrochemical properties based on considerable efforts.^13^

**Table 1:** Fundamental properties of selected transition metal carbides/nitrides. Values are for room temperature and stoichiometric composition. Reproduced with permission from ref 14

Compound	Density [g cm^−3^]	Microhardness [GPa]	Melting point [°C]	Young's modulus [GPa]	Heat conductivity [W m^−1^ K^−1^]	Linear thermal expansion coefficient [(10^−6^) K^−1^]	Electrical resistivity [μΩ cm]
Carbides							
TiC	4.93	28	3067	450	28.9	8.5	100
ZrC	6.46	25	3420	350	24.6	7.5	75
HfC	12.3	20	3930	420	25.1	6.1	67
VC_0.88_	5.36	26	2650	430	26.8	7.2	69
NbC	7.78	18	3610	340	27.0	6.6	20
TaC	14.48	16	3985	290	22.1	6.3	15
Cr_3_C_2_	6.68	27	1810	380	14.0	10.3	75
Mo_2_C	9.18	17	2520	530	15.0	7.8	57
WC	15.72	23	2776	707	19.0	3.9	17
Nitrides							
TiN	5.39	17	3050	420	29	9.9	27
ZrN	7.32	15	3000	460	11	7.8	24
HfN	13.83	18	3330	380	11	8.5	27
VN	6.04	5.7	2350	380	11	10.8	65
NbN	8.16	11	Decomposes	360	3.8	10.2	60
TaN	15.9	32	Decomposes	–	–	8.0	–
CrN	6.14	13	Decomposes	450	11.7	–	640

Group IVB–VIB transition metal carbides and nitrides are often referred to as “interstitial alloys,”^15^, ^16^, ^17^, ^18^ which are prepared by integrating carbon or nitrogen atoms into the interstitial sites of their parent metals.^19^ Transition metals are intensively capable of forming carbides, exceptionally with the Pt‐group metals. It is worth noting that iron, cobalt, and nickel are particular cases, which form iron carbide/nitride,^20^ cobalt nitride,^21^ and nickel nitride,^22^ respectively. It is indicated that in the early transition metals the formulas MX and M_2_X are prevalent, in the later transition metals the stoichiometry shifts to M_3_X.^23^

**Figure 1:**
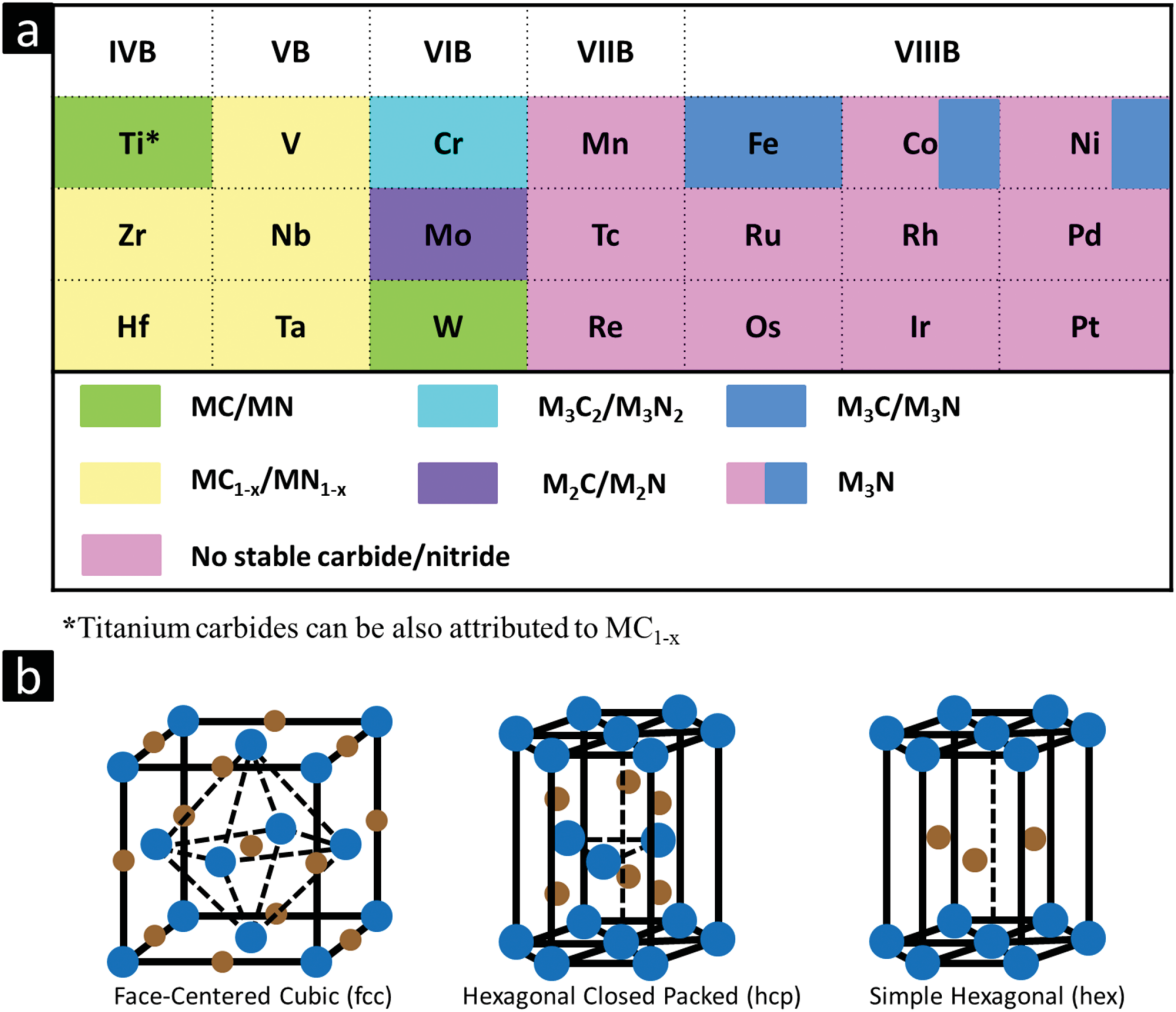
a) Typical transition metals in the TMCs and TMNs compounds. Reproduced with permission.^24^ Copyright 2013, American Chemical Society. b) Common crystal structures in TMCs and TMNs compounds. The blue points represent transition metal atoms and the brown points represent carbon/nitrogen atoms.

**Figure** **1**a displays the Group 4–10 metals and their corresponding stable carbides. In addition, their common structures, such as face‐centered cubic (fcc), hexagon‐closed packed (hcp), and simple hexagonal structures, are basically formed by the small carbon and nitrogen atoms to occupy interstitial positions (see Figure 1b).^25^ This sort of geometric phenomenon was pioneered and formulated according to the Hägg's rule:^15^ the crystal structure forms depending on the radius ratio *r* = *r*_x_/*r*_m_, where *r*_x_ and *r*_m_ are the radii of nonmetal atoms and parent metal atoms, respectively. When the *r* is less than 0.59, the metallic arrangement forms simply the common crystal structures. For the carbides and nitrides of Ti, V, Mo, and W, the radius ratios range from 0.491 (*r*_N_/*r*_Ti_) to 0.576 (*r*_C_/*r*_V_). Additionally, Houston^26^ indicated that natural structure of the parent metal could be modified by intercalation of carbon/nitrogen atoms. The metal lattice expands and the distance between metal atoms increase during formation of interstitial TMCs/TMNs, which would result in a broadening of the metal *d*‐band. Such a *d*‐band contraction would cause a greater density of states (DOS) near the Fermi level in comparison with the parent metal.^27^ It is believed that the redistributions of the DOS in turn gives rise to the catalytic properties resembling noble metals.

Continue with the previous understanding, TMCs and TMNs compounds possess combination of three different interactions between atoms: covalent bond, ionic bond, and metallic bond.^20^, ^28^ The covalent character is displayed by the extreme hardness and brittleness of these compounds, which result in a better tolerance to the stress caused by volume expansion during lithiation and delithiation process and therefore keep their structure stable. This persistent mechanical integrity has not been observed in other transition metal compounds. Mechanically robust TiC/C nanofibers^29^ were developed to enhance the conventional Si anodes, which relieved mechani­cal fracture arising from large volume expansion during the alloying reaction of Si and resulted in great improvement of cycling stability. Meanwhile, they also possess good ionic conductivity of ionic crystals and excellent electronic properties of transition metals. A very successful combination of good ionic conductivity and high electronic conductivity is achieved in one system, which is desirable for EES application. For instance, in the Li‐ion battery (LIB) system, there are three dynamic processes for Li^+^ transportation: Li^+^ motion through the electrolyte accompanied by electron flowing in the outer circuit; interfacial ion transfer; and chemical diffusion accompanied by electron in the bulk of electrode material.^27^ The diffusion of Li^+^ and electron in the electrode material is greatly affected in terms of the ionic and electronic conductivity. Additionally, TMCs and TMNs generally have good chemical stability; they are not easily eroded by dilute acids (except for oxidizing acids and hydrofluoric acid) or alkaline solutions.^14^

TMCs and TMNs, being interstitial alloys of carbon and nitrogen atoms, differ substantially from other transition compounds such as transition metal dichalcogenides (TMDs) and transition metal oxides (TMOs). TMDs have a lamellar structure similar to that of graphite, and have been investigated as electrode materials for lithium ion batteries because Li^+^ ions can easily intercalate or extract from these materials. But the electrical conductivity of TMDs, however, is too low for their effective implementation as electrodes (10^−3^ Ω^−1^ cm).^30^ Calculation shows that the elastic constant of Ti_2_C is almost twice higher than that of MoS_2_, which indicated that the TMCs have better mechanical properties. TMOs are also widely studied as suitable candidates for electrode materials of LIBs and SCs due to their high theoretical capacities (e.g., RuO_2_: 2000 F g^−1^, MnO_2_: 1370 F g^−1^). Compared to TMCs and TMNs, the synthesis of TMOs generally took place at relative low temperatures and a wide variety of tailored size and morphology can be achieved by controllable synthesis methods. However, similar to TMDS, TMOs usually have relatively low electrical conductivity, and the usage of some noble transition metals is not cost effective. When compared to Pt‐group metals, the advantageous benefits in the cost, natural abundance of the raw transition metals, outstanding thermal stability, and consistent tolerance to common catalyst poisons, make TMCs different and alternative to the noble metals. Along with all the properties mentioned above, together with the advantages against other materials, make TMNs a promising choice for the electrode material of EES devices.

For EES application of metal carbides, recent development was made by the Gogotsi group,^31^ who found a new 2D family member, “MXene” phase. MXene was found to be suitable electrode materials for supercapacitors and demonstrated high volumetric capacitances up to 900 F cm^−3^ and excellent cycling life and high‐rate capability. These exceptional properties render “MXene” advantages against other 2D materials such as graphene, TMDs, and the recently discovered silicene, germanene, and phosphorene.^32^ The most recent occurrence of EES development (LIBs) was reported by the Zhu et al.,^33^ who prepared molybdenum carbide@carbon nanosheet (CNS) composite material with remarkable lithium ion storage properties. Similarly, another rising star, TMNs have attracted wide great interests as cost effective alternatives for substituting noble metals toward a wide range of applications. Lu et al. have prepared TiN^31^ and VN^34^ as the supercapacitor electrodes materials, both of which showed encouraging capacity and excellent stability. In the field of electrochemical catalysis, Chen and co‐workers have investigated a series of catalytic TMNs^35^, ^36^ and they developed a bio‐template method^35^ by using soybeans to produce MoN for hydrogen evolution reaction (HER) application. Here, we present **Table** **2** to summarize the properties, applications, and synthesis methods of group IV–VI metal carbides and nitrides as active materials for EES and electrocatalysis in recent years.

**Table 2:** Properties, applications, and synthesis methods of TMCs and TMNs. Specific surface area, oxygen reduction reaction, oxygen evolution reaction, hydrogen evolution reaction, methanol oxidation reaction, dye‐sensitized solar cell, chemical vapor deposition, chemical bath deposition, metal organic framework and atomic layer deposition are abbreviated as “SSA,” “ORR,” “OER,” “HER,” “MOR,” “DSSC,” “CVD,” “CBD,” “MOF,” and “ALD,” respectively

Materials	Synthesis method	Morphology	Applications	Performance	ref.
Ti*_x_*C*_x_*_−1_T_n_* (MXenes)	Etching	2D Layered nanosheet	Supercapacitors Li‐ion batteries	900 F cm^−3^ or 245 F g^−1^ 225 mAh g^−1^ at C/25	31, 37, 38, 39, 40
TiC	Co‐reduction	Hollow nanospheres	High SSA	78.30 m^2^ g^−1^	41
TiC	Hydrothermal	Nanowires	ORR	85% retention	42
TiC	Sol–gel	Nanoparticles	High SSA	267 m^2^ g^−1^	43
TiC–SiC–C	Sol–gel	Nanoporous	DSSC	5.7% conversion efficiency	44
TiC	Template	Nanowires	MOR	348.3 mA mg_pt_^−1^ at 50 mV s^−1^	45
TiC/NiO	Template	Core–shell nanorods	Li‐ion batteries	568.1 mAh g^−1^ at 200 mA g^−1^	46, 47
TiC, TaC	Carbothermal reduction	Nanowires	High SSA	–	48
TiC	CVD	Nanotubes	Supercapacitors	185 F g^−1^ at 2 A g^−1^	49
TiC@ppy	CBD	Core–shell nanoparticles	Li‐ion batteries	65% retention at 20C	50
MoC@CNS	Template	Vertically aligned nanosheet	Li‐ion batteries/HER	1010 mAh g^−1^ at 200 mA g^−1^	33
WC@CNS				220 mV at 10 mA cm^−2^	
MoC/MoN	Urea glass route	Nanoparticles	HER	176 mV at 10 mA cm^−2^	51
MoC	Urea glass route	Nanotubes	DSSC	6.22% conversion efficiency	52
MoC*_x_*	MOFs‐assisted strategy	Nano‐octahedrons	HER	142 mV at 10 mA cm^−2^ in acid	53
				151 mV at 10 mA cm^−2^ in alkali	
Mo_2_C	CBD/pyrolysis	Nanowires	HER/ORR	–	54, 55, 56
Mo_2_C	In situ carburization	Nanoparticles	HER	63 mV at 10 mA cm^−2^	57
α‐W_2_C/WN@graphene	In situ carbothermal	Nanoplates	HER	120 mV at 10 mA cm^−2^	36
WC	Carbothermal reduction	Nanoparticles	HER	200 mV at mA cm^−2^	58
WC@GC	Carbothermal reduction	Nanoparticles	MOR	1191.8 mA mg_pt_^−1^ at 50 mV s^−1^	59
CoWC	Carbothermal reduction	Nanoparticles	HER	200 mV at 10 mA cm^−2^ in acid	60
MoS_2_/WC/RGO	Carbothermal reduction	Nanoparticles	HER	200 mV at 10 mA cm^−2^ in acid	61
WC	Carbothermal reduction	Nanowall	High SSA	–	62
WC/C	Coating/carbothermal	Nanoparticles	HER	200 mV at 10 mA cm^−2^ in acid	63
WC	Template	Nanoparticles	High SSA	138 m^2^ g^−1^	64
VN	Magnetron sputtering	Nanoparticles	Lithium batteries	800 mAh g^−1^	65
VN	Ammonolysis	Nanoparticles	Supercapacitors	554 F g^−1^ at 100 mV s^−1^	66
VN	Ammonia reduction	Nanosheets	Supercapacitors	1937 mF cm^−3^	67
VN/CNT	Sol–gel/ammonia reduction	Nanoparticles	Supercapacitors	270 F g^−1^	68
TiN	CVD	Film	–	–	69, 70, 71, 72
Ni*_x_*Co_2*x*_(OH)_6*x*_/TiN	Anodization/ammonia/electrodeposition	Nanotube arrays	Supercapacitors	2543 F g^−1^ at 5 mV s^−1^, 660 F g^−1^ at 500 mV s^−1^	73
MnO_2_/TiN	Anodization/ammonia/electrodeposition	Nanotube arrays	Supercapacitors	681 F g^−1^ at 2 A g^−1^, 267.2 F g^−1^ at 2000 mV s^−1^	74
TiN/CNT	Hydrothermal/ammonia reduction	Nanotubes	DSSCs	5.41% conversion efficiency	75
TiN@C	Hydrothermal/ammonia reduction	Nanowires	Supercapacitors	124.5 F g^−1^ at 5 A g^−1^	31
TiN@GNS	ALD	Vertically aligned nanosheet	Supercapacitors	0.51 mWh cm^−3^ energy density	76
FeN@GNS				211.4 mW cm^−3^ power density	
WN	CVD	Film	–	–	77, 78
WON	Hydrothermal/ammonia reduction	Nanowires	Supercapacitors	1.27 mWh cm^−3^ energy density	79
				1.35 W cm^−3^ power density	
Mo_2_N@RGO	Bio‐template	Nanoparticles	HER	109 mV at 10 mA cm^−2^ in acid	35
Mo_2_N@GNS	Hydrothermal/ammonia reduction	Nanosheets	Li‐ion batteries	428 mAh g^−1^ at 100 mA g^−1^	80

In view of the thriving of TMCs and TMNs materials, in this article, we will review the recent progress made in TMCs and TMNs based electrodes in EES (e.g., LIBs, SIBs, and supercapacitors) and electrocatalysis [oxygen reduction reaction (ORR), HER, and oxygen evolution reaction (OER)]. The article starts with synthesis methods for these materials, and then elaborate in great details the electrochemical performance of various TMC and TMN electrode materials in a few types of batteries and electrocatalytic reactions. At the end, we will give an overall conclusion and our outlook to the future direction in the TMCs and TMNs for EES and electrocatalysis.

## 2 Synthesis Methods for Transition Metal Carbides and Nitrides

It is well known that the performance of both fuel cells and EES devices are highly dependent on the reactivity of electrocatalysts and electrode materials. As a consequence, design and fabrication of high‐quality materials become the fundamental step before constructing high‐performance electrodes. Up to now, great progress has been achieved for fabrication of nanostructured TMCs and TMNs with enhanced electrochemical performance. Tailor‐made nanostructures are demonstrated with increased specific surface areas and larger reaction sites as well as sufficient contact area between reactants/electrolyte and active materials. These favorable characteristics endow TMCs and TMNs with much improved catalytic/electrochemical properties, which push their practical applications especially in catalysis and energy storage areas. In the following section, we will summarize and discuss the recent progress in the synthesis methods of TMCs and TMNs and their composite materials.

### 2.1 Titanium Carbides

Titanium carbide, a typical representative of TMCs materials, has an extremely high melting point up to 3260 °C, high rigidity, high electrical conductivity, and exceptional chemical and thermal stability.^81^, ^82^, ^83^ The fluctuations of C:Ti stoichiometry ratios do not affect the stability of its cubic system symmetry (Fm3m) structure.^84^, ^85^ The traditional/common synthetic method for titanium carbide is direct solid‐state reaction (also called solid‐state carbothermal method), in which metals (or metal oxides) and elemental carbon (or other solid carbon materials) react with each other at high temperature between 1500 and 2300 K. Note the fact that this solid‐state reaction temperature is higher than other carbides due to the higher activation energy for the formation of titanium carbide. Moreover, the high temperature accelerates the solid‐state diffusion of carbon into metal‐oxides or metals to from TiC.^86^, ^87^ The involved solid‐state reactions can be simply described as follows

(2.1) Ti (s)+C (s)  →  TiC (s)

(2.2) TiO (s)+2C (s)  →  TiC (s)+CO (g)

(2.3) $${\mathrm{TiO}}_{2}\;(s)+2C\;(s)\;\;\to \;\;\mathrm{TiC}\;(s)+{\mathrm{CO}}_{2}\;(g)$$

Meanwhile, the TiC can also be prepared by gas–solid reaction via thermal decomposition of gas precursors (such as CH_4_ and C_2_H_2_) at the surface Ti or TiO_2_ 23, 88

(2.4) $$\mathrm{Ti}\;(s)\;+\;{\mathrm{CH}}_{4}\;(g)\;\;\to \;\;\mathrm{TiC}\;(s)\;+\;2H_{2}\;(g)$$

Generally, the TiC products prepared by gas–solid approaches are dense micrometer sized particles with low surface area (<10 m^2^ g^−1^)^89^, which is mainly affected by the size and diffusion direction of Ti and carbon resources during the formation of TiC.^90^ As the advancement of nanotechnology, the last decades experience the rapid change in the innovation of synthesis methods of nanostructured TiC materials. A typical example could be found in Qian and co‐workers,^91^ who developed a low‐temperature co‐reduction method to prepare TiC particles of 10–20 nm by sodium co‐reducing TiCl_4_ and CCl_4_ at 450 °C. The reactions involved in the co‐reduction process could simply be represented as follows

(2.5) $${\mathrm{TiCl}}_{4}\;(l)\;+\;C_{2}{\mathrm{Cl}}_{4}\;(l)\;+\;8\mathrm{Na}\;(s)\;\;\to \;\;2\mathrm{TiC}\;(s)\;+\;8\mathrm{NaCl}\;(s)$$

The key of this method is the element of Na, which acts as a strong reluctant, and enables the Ti and C to react with each other forming TiC nanoparticles (NPs).^92^ This co‐reduction method is further modified by Zhong et al.,^43^ who utilized sol–gel and carbothermal reduction process to fabricate nano TiC grains (30–50 nm) with a high surface area of 267 m^2^ g^−1^. It is reported that the surface area will be further improved when TiC is composited with other materials such as carbon nanotubes (CNTs)^93^ and graphene.^94^ Impressively, Yu et al.^95^ have achieved continuous progress and reported the synthesis of TiC‐carbon composites by using a solvent‐evaporation‐induced self‐assembly approach. TiC nanocrystals with size of 4–5 nm were obtained and embedded into the mesoporous carbon matrix by the carbothermal reduction of titanium citrate at above 1000 °C. Their samples have a high surface area up to 823 m^2^ g^−1^.

**Figure 2:**
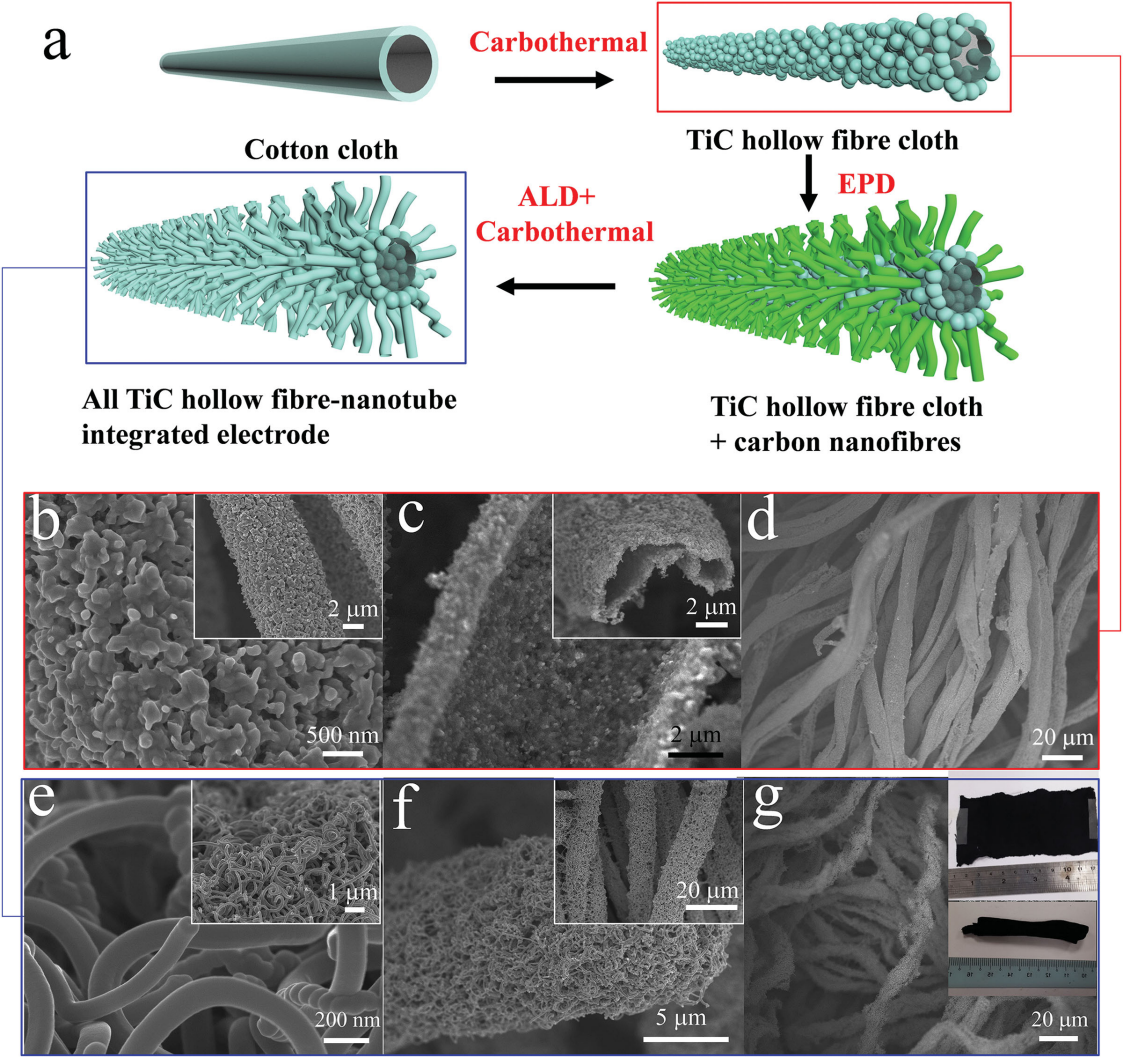
TiC hollow fibers branched with nanotubes made from commercial cotton T‐shirt and used for supercapacitor electrodes. a) Schematics of the structure and fabrication process of the branched hollow fibers. b–d) Scanning electron microscope (SEM) images of TiC hollow fiber (without tube branches) cloth in different magnifications. e–g) SEM images of TiC hollow fiber with nanotube branches. Photos of the TiC cloth are presented in (g). Reproduced with permission.^49^ Copyright 2015, Royal Society of Chemistry.

Currently, it still remains a challenge to synthesize nanostructured metal carbides with tailored dimension and morphology because of the inhomogeneous carburization and inevitable aggregation at high reaction temperatures. In this context, the template synthesis method emerges as the times require. Compared to other methods, template method is much more preferable for the preparation of nanostructured metal carbide materials due to the fact that the structure, morphology, and size of metal carbides can be controlled by simply altering the nature of template and the preparation conditions.^45^, ^46^, ^48^, ^96^, ^97^ Interesting 1D or 2D morphologies (such as nanowires, nanorods, nanowires, nanotubes, and nanosheets) could be obtained by using corresponding templates. Among the template methods, a growing number of investigations have been devoted to bio‐templates, which are based on the natural species such as cottons,^48^, ^98^ bamboo,^48^, ^99^ and aquatic plants,^100^ offering special exterior and interior surfaces and uniform geometries. More recently, our group developed an atomic‐layer‐deposition (ALD) assisted template method for fabrication of TiC tubular fibers, and hollow TiC nanotube, and their composites (**Figure** **2**).^49^ Commercial cotton T‐shirt and carbon nanofibers were used both as the carbon source and sacrificial template. Ni catalyst (from the reduction of nickel nitrate) was also adopted to accelerate the carbothermal reaction. Cotton had a striking hydrophilicity that absorbs easily polar solvents (e.g., water and ethanol). Therefore, when the cotton was soaked in the precursor solution, it is possible that the cotton fully absorbs the solution with titanium source and catalyst precursor. This is not trivial because it assures a thorough reaction, homogeneous composition of the final structure and shape reservation of the fiber structure. The obtained TiC hollow fiber cloth and nanotubes showed a reasonably good conductivity (≈10^5^ S m^−1^) and good flexibility. The reactions toward the formation of TiC micro/nanofibers are deemed not straightforward. The possible reactions could be proposed as follows

(2.6) $$\mathrm{NaF}\;(l)\;\;\to \;\;\mathrm{Na}\;(g)+F_{2}\;(g)$$

(2.7) Ni@C (s)  →  Ni(C) (l)

(2.8) $${\mathrm{TiO}}_{2}\;(s)+xF_{2}\;(g)+4C\;(s)\;\;\to \;\;2{\mathrm{TiF}}_{x}\;(g)+4\mathrm{CO}$$

(2.9) $$2{\mathrm{TiF}}_{x}\;(g)+2\mathrm{Ni}(C)\;(l)\;\;\to \;\;2\mathrm{Ni}(\mathrm{Ti},C)\;(l)+xF_{2}\;(g)$$

(2.10) Ni(Ti,C) (l)  →  TiC (s)+Ni (l)

**Figure 3:**
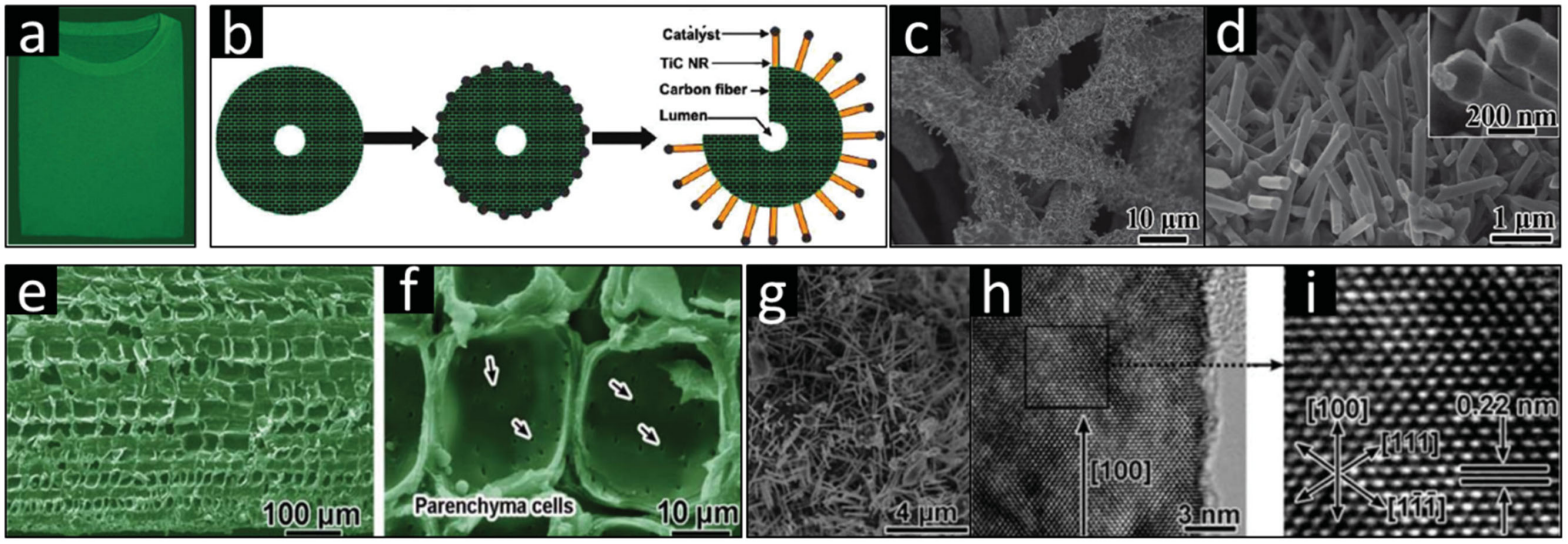
a–d) TiC nanorods array on carbon fiber made from commercial cotton T‐shirt. c,d) SEM images of the nanorod arrays. e–i) TiC nanorods obtained from bamboo culm. e–f) Typical SEM image of the porous bamboo culm. g–i) Electron images with increasing magnifications of a TiC nanowire. Reproduced with permission.^48^ Copyright 2011, Royal Society of Chemistry.

Additionally, Tao's group prepared titanium carbide nanowires and nanorods by using commercial cotton T‐shirt (**Figure** **3**a) and natural nanoporous bamboo (Figure 3e) as carbon source and template, respectively.^48^ Both of the samples were synthesized by a two‐step process: the bio‐templates and titanium source were first mixed somehow and then heated at about 1000–1350 °C under argon protection. The vapor–liquid–solid (VLS) mechanism has been proposed for the growth of the 1D carbides, which is a well‐established mechanism in catalyst‐guided growth of nanowires. The TiC samples produced from bamboo were mainly composed of straight nanowires with typical length larger than 2 mm and the nanorods produced from cottons had a dia­meter varying from 80 to 200 nm and length ranging from 1 to 3 μm. These nanostructures are formed due to the natural porous bio‐templates. Furthermore, the process is effective and the raw materials are low‐cost, allowing the large‐scale production of titanium carbide to meet the high demand of industry.

Except for the bio‐templates, other TiC nanostructures including nanospheres, nanowires, and nanotubes have also been successfully prepared by templates such as carbon nanotube and sea wool sponge template. The morphology of these templates is well kept after high‐temperature annealing process, and high surface areas are realized in these samples.

Core/shell nanoarchitecture has been an effective design for TiC based composite materials, in which the advantages of both components can be more effectively utilized toward functional enhancement.^29^, ^101^, ^102^, ^103^, ^104^ Cui group pioneered a one‐step chemical vapor deposition (CVD) method for the fabrication of TiC/C core/shell nanowire arrays on Ti_6_Al_4_V alloys substrate by adopting acetone as the carbon sources.^29^ The carbon shell was well coated on the surface of TiC forming core/shell composite arrays. However, the specific growth mechanism is not clear. It was believed that the Al and V in the alloys have catalytic effect for the preferential growth of TiC/C core/shell arrays; no core/shell nanowires formed on the pure Ti substrate under the same deposition condition. Furthermore, the as‐prepared TiC/C core/shell nanowire arrays could serve as highly conductive and strong backbone for the growth of Si or Fe‐Fe_3_O_4_ and other active materials for EES application. Zhang's group reported the construction of TiC/NiO core/shell nanostructures on TiC nanowires skeleton as the electrode materials for lithium ion storage.^46^ Similar phenomenon happened in TiC composites prepared by cotton template. Zhang and co‐workers^46^ employed natural nanoporous cotton template to prepare TiC nanowires and TiC/Pt composites. It is found that the as‐prepared TiC nanowires are superior support for Pt particles with enhanced electrocatalytic performance. As a result, it is reasonable that TiC with particular nanostructure can be easily combined with other methods to fabricate metal carbides based composites. Encouragingly, in view of unique chemical/physical properties (especially high electrical conductivity and chemical stability), these composites usually show enhanced optical/electrical properties than those unmodified ones.

The 2D concept has also extended to TMCs family. Recently, MXenes as a new type of layered materials have been reported by the Gogotsi group.^38^, ^40^ The MXenes were produced by selectively etching aluminum off titanium aluminum carbide (Ti_3_AlC_2_, a “MAX” phase) in concentrated hydrofluoric acid. According to Gogotsi, MAX phases are a family of more than 60 members of layered ternary transition metal carbides or nitrides, where M stands for an early transition metal, A is a group IIIA or IVA element, and X is C and/or N. These 2D materials obtained by etching MAX phases are named as “MXenes” because of their similarity in layered structure to graphene. The etching reactions can be illustrated as follows in the case of titanium carbide

(2.11) $$M_{2}\mathrm{AlC}+3\mathrm{HF}\;\;\to \;\;{\mathrm{AlF}}_{3}+{\mathrm{Ti}}_{2}C+1.5H_{2}$$

(2.12) $${\mathrm{Ti}}_{2}C+2H_{2}O\;\;\to \;\;{\mathrm{Ti}}_{2}C{\left(\mathrm{OH}\right)}_{2}+H_{2}$$

(2.13) $${\mathrm{Ti}}_{2}C+2\mathrm{HF}\;\;\to \;\;{\mathrm{Ti}}_{2}{\mathrm{CF}}_{2}+H_{2}$$

According to the above reactions, the surface of 2D Ti_2_C contains OH^−^ or F^−^ groups, which explains the presence of O and F after treatment. The selective etching process could also be applicable to the fabrication of transition metals such as Ta, Nb, and V. Those 2D materials have shown an exceptional combination of metallic conductivity, surface hydrophilicity, and also energy storage functionalities. But their physicochemical properties are limited by defects and surface terminal groups due to lack of large and high‐quality crystals. In this regard, Xu et al.^105^ synthesized 2D molybdenum carbide by CVD method from methane on a substrate of copper and molybdenum foil. The as‐synthesized samples have perfect crystalline quality with little observable defective, making them structurally robust and chemically stable.^32^ The mechanical properties were studied by using large‐scale classical molecular dynamics simulations.^106^ Although the Young's modulus of MXenes are almost twice smaller than thin graphene (1.0 TPa), the elastic constant of Ti_2_C (0.597 TPa) is almost twice higher than MoS_2_ (0.33 TPa). Very recently, Anasori et al.^107^ predicted the existence of stable double transition metals carbides using density functional theory (DFT). Two main trends for the stabilities of this kind of MXenes are identified: One is that metal elements whose binary carbides do not form in the rock salt structure, like Mo and Cr, avoid the center layers. Another is for Nb and Ta, they prefer the middle layers. Continuous with their investigations, Mo_2_TiC_2_T*_x_*, Mo_2_Ti_2_C_3_T*_x_*, and Cr_2_TiC*_x_*T*_x_* were then synthesized by simply etching in HF. This work significantly enlarged the MXene family and it is expected that in the future, other forms of MXene materials such as nanotubes can be discovered as well.

### 2.2 Molybdenum Carbides

Over the past decades, molybdenum carbide is one of the most widely studied transition metal carbides in the light of its intrinsic high catalytic activity similar to Pt‐group metals, low cost, high abundance, and good electrical conductivity.^108^ It is well known that molybdenum carbides have four different crystal structures (i.e., α‐MoC_1−*x*_, β‐Mo_2_C, γ‐MoC, η‐MoC^109^ with variable compositions, which will greatly affect the electrochemical performance. The different crystal structures not only change the dispersion amount of Mo and C at the surface to affect the catalytic properties but also have different tunnels size influencing the insertion of ions. α‐MoC_1−*x*_ possesses the same crystal structure as NaCl with fcc structure. The other molybdenum carbides (β‐Mo_2_C, γ‐MoC, and η‐MoC) have similar hexagonal crystal structures but their stacking sequences are different.^110^, ^111^

Different synthesis conditions can lead to various molybdenum carbide structures, among which α‐MoC_1−*x*_ 112, 113 and β‐Mo_2_C^114^, ^115^, ^116^ are more attractive due to their higher electrochemical properties. The electrochemical properties of molybdenum carbide are highly associated with their surface areas, morphology, and surface composition, which are closely related with the synthesis method. So far, a few approaches are available for the synthesis of molybdenum carbide nanostructures. Most of these methods are connected to reductive carburization of molybdenum oxide particles in the CH_4_–H_2_ mixture,^117^, ^118^, ^119^ including temperature‐programmed reaction^109^ and CVD,^120^ pyrolysis of metal complexes,^55^ liquid‐phase reaction,^56^ template method,^33^ etc. One of the most common methods is the temperature‐programmed reduction (TPR) of the molybdenum oxide by gaseous hydrocarbons, as pioneered by Levy and Boudart. It is a gas–solid reaction between oxides and the mixture of hydrogen and carbon‐containing gases including CH_4_, C_2_H_6_, C_3_H_8_, and C_4_H_10_.^121^, ^122^ They reported the synthesis of molybdenum carbide (α‐MoC_1−*x*_) with relatively high surface areas of 150 and 185 m^2^ g^−1^. However, after a thermodynamic analysis, it was found that the obtained molybdenum carbides could be contaminated with chars that were difficult to remove. Chars blocked the pores and covered the active sites of the metal carbide. Furthermore, the rigorous and tedious synthetic conditions result in a low‐efficiency production of molybdenum carbides.^56^

To further improve the density of surface active sites of molybdenum carbides, Chen et al.^57^ conducted in situ carburization to construct covalently anchored β‐Mo_2_C nanoparticles (particle size 7–15 nm) on CNTs. The involved reactions can be illustrated as follows

(2.14) $${\left({\mathrm{NH}}_{4}\right)}_{6}{\mathrm{Mo}}_{7}O_{24}\cdot H_{2}O(130\;\;{}^{\circ}C)\;\;\to \;\;7\alpha {\mathrm{‐MoO}}_{3}+6{\mathrm{NH}}_{3}+4\;\;H_{2}O$$

(2.15) $$2\alpha {\mathrm{‐MoO}}_{3}+7C(800\;\;{}^{\circ}C)\;\;\to \;\;\beta {\mathrm{‐Mo}}_{2}C+6\mathrm{CO}$$

The as‐fabricated Mo_2_C nanoparticles were firmly anchored on the carbon support, which builds up a fast electron transfer path. Followed with another work about Mo_2_C‐graphite, Yan et al. prepared extreme thin particles of Mo_2_C‐graphite with sizes of 2 nm based on an ion exchange process.^123^ In this approach, the graphite layers and Mo_2_C were formed at the same time because the growth of Mo_2_C and graphite layer would take place simultaneously in the specified temperature range.

Some two‐step liquid‐phase reactions have also drawn attentions. The advantage of liquid‐phase reactions is that they can generate nanoparticles with high crystalline quality and purity.^51^ Giordano et al.^124^ have developed a soft urea glass liquid‐phase approach for preparation of metal carbides and nitrides. Compared to other methods, the urea glass method requires much lower temperature and its processing is more facile. Based on this method, Wu^124^ and Ma^51^ prepared Mo_2_C nanotubes and Mo_2_C/Mo_2_C nanoparticles, respectively, both of which showed high crystallinity and uniform nanoscale size.

**Figure 4:**
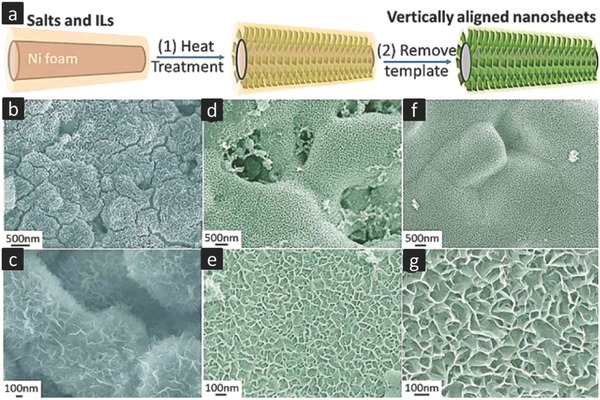
MoC_0.654_@carbon composite nanosheet obtained via a salt‐templating process. a) Schematics of the formation process. b,c) SEM images of the nitrogen graphitic carbon nanosheets, d–g) Two examples of the obtained nanosheets array of MoC_0.654_@CNS composites. Reproduced with permission from ref. 33. Copyright 2015, American Chemical Society.

Ordered and controllable molybdenum carbide nanostructures are still highly desirable. A novel and efficient approach was applied by Zhu et al. to fabricate vertically aligned 2D carbon nanosheets and MoC_0.654_ based on a simple salt template method.^33^ The authors used graphitic CNS as the skeleton for the deposition of MoC_0.654_ to form composite nanosheets (see **Figure** **4**). Such vertical aligned composite nanosheets are highly conductive and porous, with a good structural robustness. A large electrode/electrolyte contact and short ion diffusion herein are favorable for high‐rate energy storage and activity catalysis.

**Figure 5:**
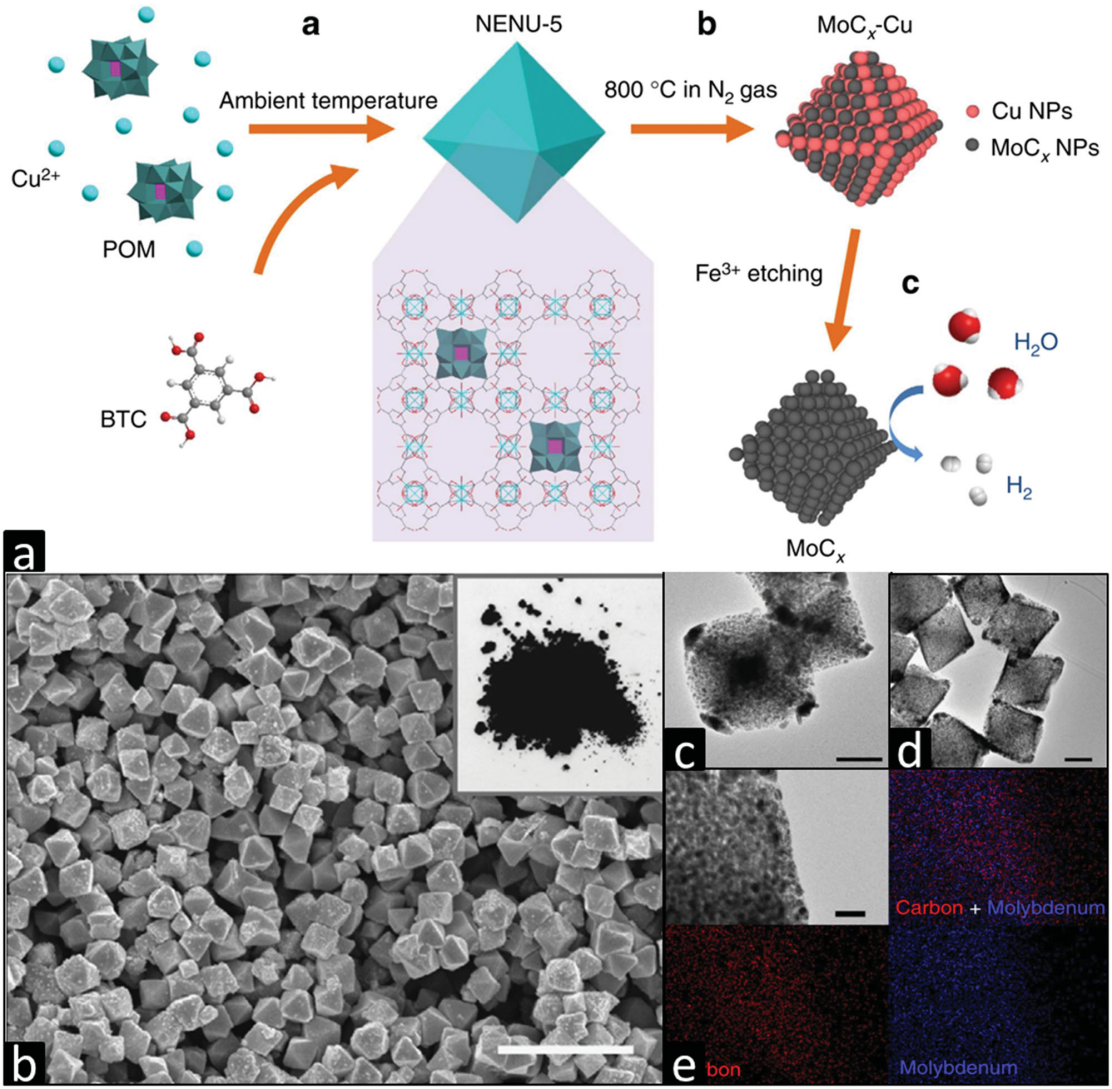
Porous MoC*_x_* nano‐octahedrons obtained from MOF template. a) Schematics of the synthesis procedure; b) SEM image of the porous MoC*_x_* nano‐octahedrons (scale bar, 2 μm); c–e) TEM images and EDX elemental mappings of the MoC*_x_* octahedrons. Scale bars are 200 nm in (c) and (d), and 50 nm in (e). Reproduced with permission.^53^ Copyright 2015, Nature Publishing Group.

The highly porous and ordered structure of metal–organic frameworks (MOFs) provides particular advantages for the preparation of nanostructured metal carbides, which always exhibit large surface areas and high porosities as inherited from the porous MOFs template. A brilliant work conducted by Wu et al.^53^ who adopted MOFs as both precursor and template for the synthesis of MoC*_x_*. During the thermal treatment, the Mo‐based guest polyoxometalates reacted with carbon sources to form MoC*_x_* nanocrystallites, and meanwhile, Cu^2+^ clusters were reduced to metallic Cu, resulting in the formation of MoC*_x_*‐Cu (see **Figure** **5**). The last step was etching the Cu particles in a FeCl_3_ aqueous solution, giving rise to the final MoC*_x_* nano‐octahedrons that comprises ultrafine nanocrystallites. These structure advantages bring the MoC nano‐octahedrons remarkable catalytic performance for HER in both basic and acidic conditions.

### 2.3 Tungsten Carbides

Since the seminal work of Levy and Boudart, WC has been extensively studied as superior Pt‐like catalysts,^12^ and proven with high activities similar to those of noble metal catalyst.^125^, ^126^ Tungsten carbide has a high melting point at 2870 °C, a boiling point of 6000 °C at a pressure equivalent to 1 standard atmosphere (100 kPa), a thermal conductivity of 110 W m^−1^ K^−1^, and a thermal expansion coefficient of 5.5 μm m^−1^ K^−1^. WC and W_2_C are two well‐characterized compounds tungsten and carbon; the proportions present in coatings depend on the specific synthesis method.^127^ Among these various tungsten carbide phases, α‐W_2_C^128^, ^129^ and β‐WC_1−*x*_ (*x* ≈ 0.5)^130^, ^131^, ^132^ attracted the most attention in catalysis. The α‐W_2_C has an hcp structure, while the β‐WC_1−*x*_ has a rock salt fcc structure. WC is thermodynamically more stable than W_2_C at low temperatures, and WC has shown its electrochemical stability in acidic solutions. For tungsten carbides like β‐WC_1−*x*_, their lattice dimension is found to increase with the carbon/metal ratio during the fabrication process. Their electronic band structures are reported to be similar to that of platinum.^133^, ^134^, ^135^ A large number of reactive facets at the surfaces is important to achieving high electrocatalysis performance for tungsten carbides.^136^ Early studies focus on traditional carburization method to produce tungsten carbides, which are based on a direct solid‐state reaction between tungsten and carbon elements, or reduction of the tungsten oxide (WO_3_) at very high temperatures of around 2000 K. Similarly, another two‐step process of WC production has also been used, namely, hydrogen reduction of oxide to high‐purity metallic tungsten followed by solid reaction with a required amount of carbon and reacts at high temperatures of 1400–1600 °C.^137^ But the obtained tungsten carbides from this method have large particle sizes and low specific surface area.

Then TPR technique is introduced into this method to produce high surface area tungsten carbides.^138^ The TPR can greatly reduce the size of products and improve the surface area of tungsten carbides. For example, the β‐WC_1−*x*_ prepared by TPR solid‐state reaction exhibited a high surface area of 49 m^2^ g^−1^ from carburization of tungsten oxides (β‐WO_3_) and CH_4_/4H_2_ mixture. Impressively, when the reactants change into β‐W_2_N and CH_4_/4H_2_ mixture, a much larger surface area of 100 m^2^ g^−1^ will be obtained.^136^, ^139^ It is indicated that the precursor have a great influence on the size and morphology of the final tungsten carbides. Additionally, ultrathin nanoparticles of tungsten carbides have been successfully synthesized by Garcia‐Esparza et al.^140^ by the solid reaction between tungsten precursors (WCl_6_) and graphitic C_3_N_4_ (mpg‐C_3_N_4_) in an N_2_ ambient. Two‐phase tungsten carbides (hex α‐WC and hcp α‐W_2_C) with sizes down to 5 nm could be obtained from this synthetic procedure by optimizing the precursor weight ratio and synthesis temperature.

After unremitting efforts, researchers have developed alternative facile approaches to fabricate tungsten carbides and their composites at lower temperatures through the carburization of tungsten oxide or other precursors,^141^ or via carbothermal reduction process, such as thermochemical spray drying method^142^ and pyrolysis of metal complexes methods.^64^ For example, Pol et al. prepared WC nanotubes by thermal decomposition of W(CO)_6_ in the presence of Mg powder at 900 °C. The obtained WC nanotubes had diameters ranging from 30 to 70 nm, and the length from 1 to 10 μm. But the fabrication procedure was tedious and difficult to scale up. In addition, wet chemical synthesis^143^, ^144^ at low temperatures are also employed for large‐scale production of tungsten carbides. Meng et al.^144^ adopted an intermittent microwave heating (IMH) method to fabricate tungsten carbides and composites by using carbon, tungsten, and H_2_O_2_ as the precursors.

Despite numerous synthesis methods for WC NPs, there are only a few that can simultaneously prevent sintering of the WC nanoparticles and mitigate surface impurity deposition. In this regard, Hunt et al.^63^ have developed a multistep method to construct high‐quality tungsten carbides and composites. They utilized spherical SiO_2_ as the template and core for coating tungsten precursors (WO_3_) to prevent WC particles sintering, but can allow the diffusion of carburization gases onto the metal oxide surfaces during annealing, and finally forming the tungsten carbides nanoparticles with sizes of 1–4 nm. This template method can effectively suppress the aggregation of WC nanoparticles and keep uniform dispersion.

**Figure 6:**
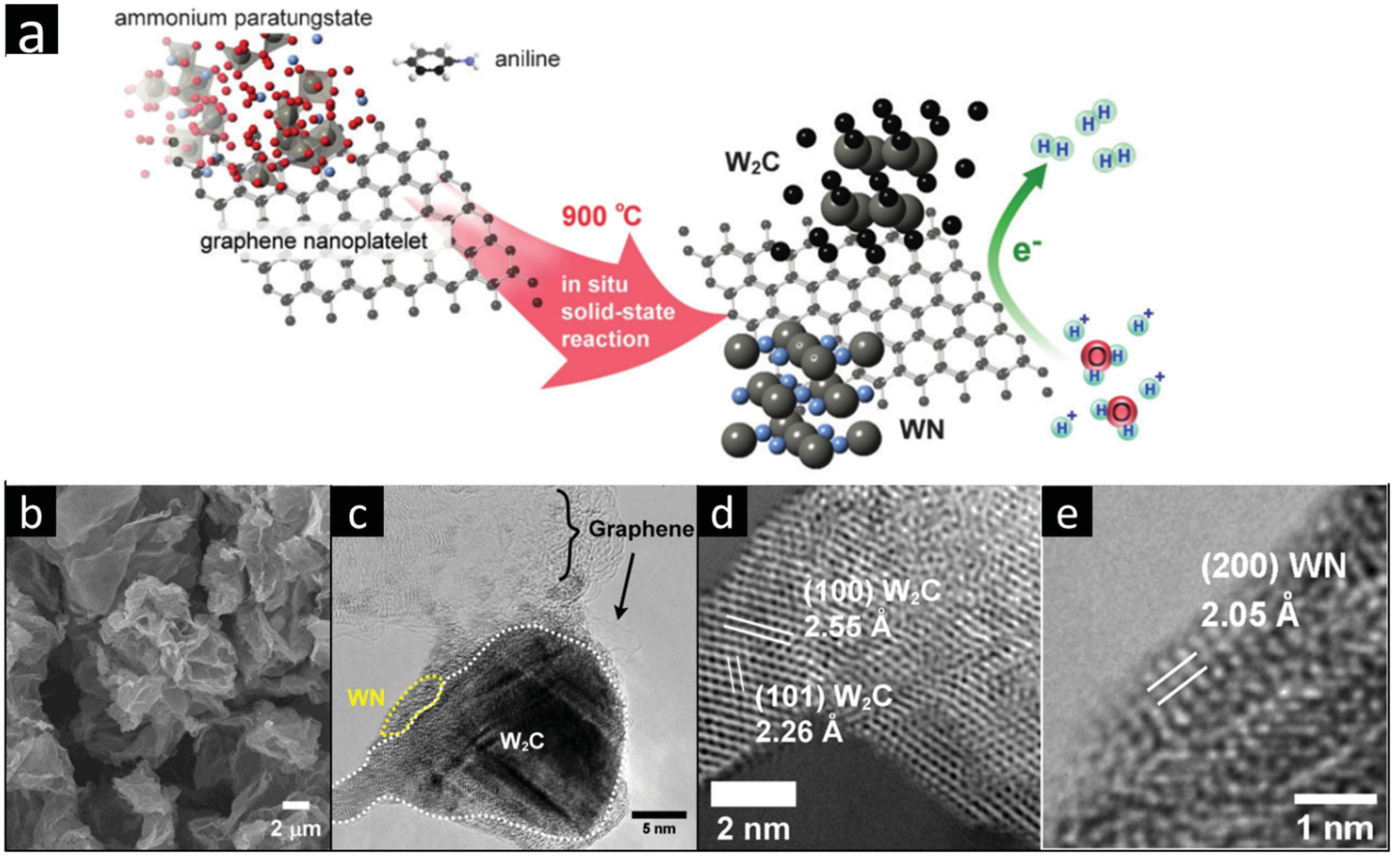
W_2_C and WN nanoparticles supported on graphene nanoplatelet. a) Schematics showing the formation pathway and structure of the composite electrocatalyst. b) SEM image and c) TEM image of the graphene nanoplatelet‐supported W_2_C and WN composite. d) High‐resolution TEM images of multilayered W_2_C and e) a WN particle in the composite. Reproduced with permission.^36^

We cannot ignore the truth that most aforementioned synthesis approaches are only for nanoparticles. Meanwhile, only a few reports are available^141^, ^145^ about the synthesis of unidirectional WC nanostructures. By now, large‐scale fabrication of pure WC nanowires or nanosheets with high structural and crystalline qualities has not been demonstrated. A straightforward way to figure out the situation that is composited with nanostructured carbon materials such as graphene^146^ and CNTs.^147^ Chen et al.^36^ reported graphene‐supported W_2_C and WN nanocomposites through a solid‐state reaction between aniline and the tungsten precursors (ammonium paratungstate, (NH_4_)_6_H_2_W_12_O_40_.*n*H_2_O). The tungsten carbide and nitride composites were attached strongly on the graphene nanoplates and shown improved electrochemical kinetics and stability compared to pure tungsten carbides (**Figure** **6**). This can be supported by another study by the Bitter group, who prepared WC/C composites using different types of carbon nanofibers. It was shown explicitly that the tungsten carbide nanostructures on carbon matrix will strongly improve the reaction pathway of catalyst,^148^ which makes a highly competitive electrocatalyst for energy production.

### 2.4 Titanium Nitrides (TiN)

TiN as a hard ceramic material is often used as a coating to metal substrates. In recent years, titanium nitride is also considered as a desirable electrode material because of their excellent reactivity and high capacity.^149^ Titanium nitride films, especially as protective coating, are conventionally prepared by various CVD techniques^69^, ^70^, ^71^ and sputtering process.^150^, ^151^ To date, some other methods are also developed to synthesize nanoscale titanium nitride particles. Su et al.^152^ have prepared a high crystallinity nano‐cubic structured titanium nitride particles by arc‐discharge method. The as‐prepared TiN nanoparticles showed a perfect cubic structure with a size of ≈50 nm.

Over recent years, the mainstream for the synthesis of TiN nanostructures is to use combined methods, that is, first to prepare the Ti‐containing precursors (such as TiO_2_) by different methods (e.g., hydrothermal, anodic anodization, sputtering), and then anneal the precursor in ammonia atmosphere, in which the precursor with Ti source will be reduced by the ammonia and converted into TiN at about 800 °C. Lu et al. prepared lots of TiN nanostructures and composites by the combination of hydrothermal and post ammonia annealing methods. First, they prepared TiO_2_ nanowires precursors on the carbon cloth by the hydrothermal synthesis and then converted them into TiN nanowires via the reduction in ammonia atmosphere. They pointed out that the degree of conversion between TiO_2_ and TiN was highly associated with the annealing time. If the annealing time is very short, there will be a thin TiN layer formed on the surface of the TiO_2_ nanowires. It is accepted that the TiN is not stable in the aqueous media or oxygen atmosphere when they are under electrochemical oxidations. TiN can be easily changed into TiO_2_ in an irreversible conversion,^153^ which will lead to a significant loss of reactivity. In order to stabilize the TiN materials, researchers combined TiN with more stable materials such as carbon materials,^154^, ^155^, ^156^, ^157^ metal oxides,^158^, ^159^ and polymers to protect the TiN from the formation of TiO_2_.^157^, ^160^, ^161^ Lu et al.^31^ prepared TiN nanowire arrays on the carbon cloth and then coated a stable amorphous carbon protective shell to their surface by using a glucose‐assisted hydrothermal method. It is proven that this shell modification is quite effective to suppress the irreversible conversion of TiN leading to enhanced cycling life.

**Figure 7:**
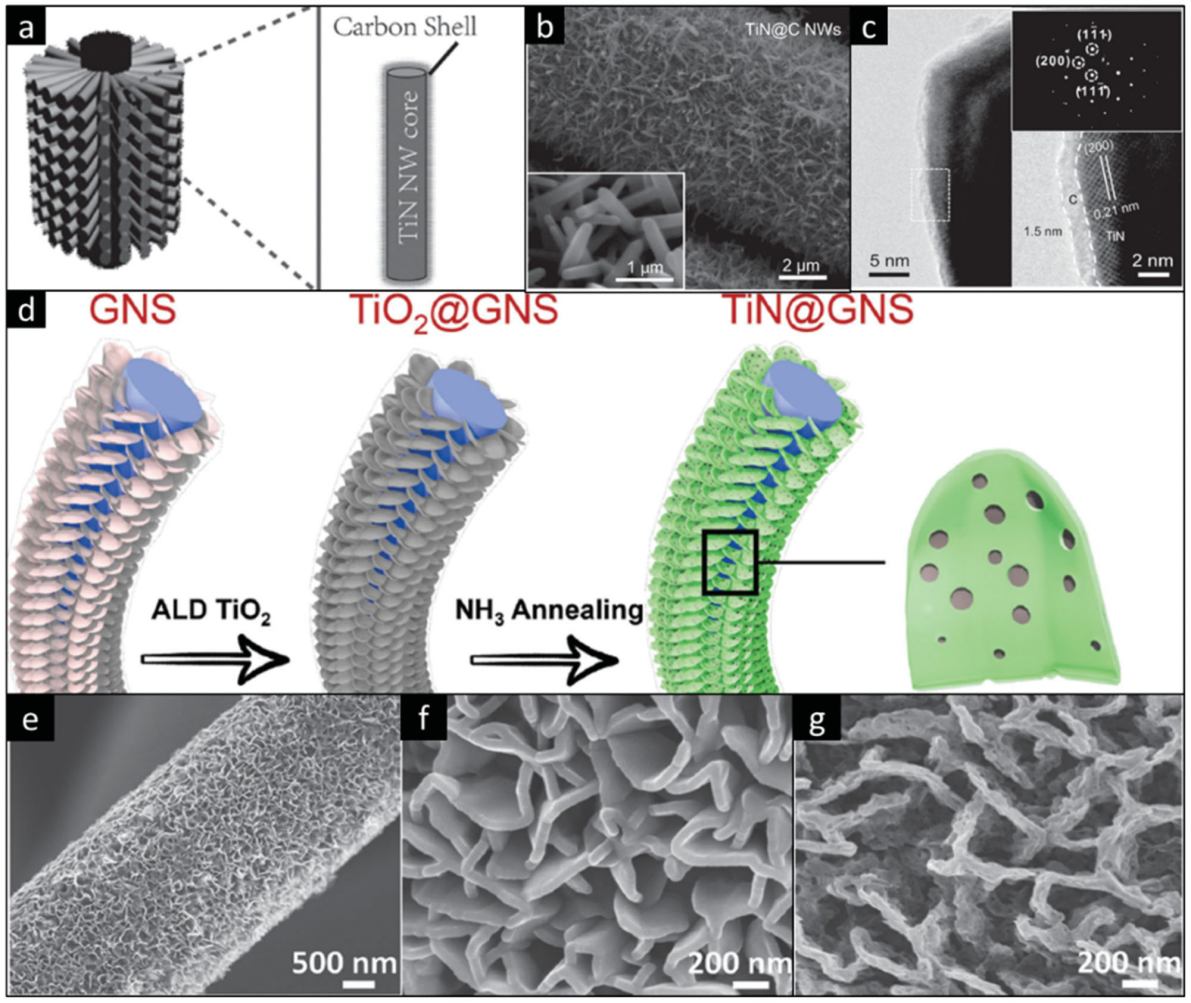
TiN‐based composite nanostructures. a) Schematics of the TiN@C core/shell nanowires array grown on a carbon fiber substrate. b) SEM image and c) high‐resolution TEM image of the TiN@C nanowire. d) Schematics of the fabrication procedure of graphene nanosheets (GNS)‐supported TiN nanoparticles. SEM images of the sample at different stages: e) GNS grown on a carbon fiber, f) ALD TiO_2_@GNS, and g) TiN@GNS. a,b) Reproduced with permission.^31^ Copyright 2014, Nature Publishing Group. d–f) Reproduced with permission.^76^

More recently, our group^76^ employed an atomic layer deposition (ALD) plus post ammonia annealing method to fabricate TiN on vertically aligned graphene nanosheets (GNSs). As illustrated in Figure 7, thin films of TiO_2_ were first coated by ALD onto the GNSs substrates and then TiO_2_@GNS samples were thermal annealed in ammonia for 1 h at 800 °C. The SEM images suggested that the smooth surface of the original TiO_2_@GNS becomes porous after the annealing process, which result in a higher surface area. Additionally, the composites showed an encouraging performance as cathode for solid‐state asymmetric supercapacitors (ASC) compared to other PVA‐based ASC devices, which is due to the combination of superior electrical conductivity of GNSs, porous structure, and high electrochemical performance of TiN nanoparticles.

Some researches indicated that the properties of metal nitrides could be further tuned by the introduction of other metal/nonmetal materials.^162^ Regarding this assumption, Li et al.^75^ introduced CNTs into TiN to improve electrical conductivity and capacitance. The precursor was obtained by hydrolysis of TiOSO_4_ on CNTs and the recovered solid was subsequently annealed under an ammonia reduction process to from TiN/CNTs composites. Hence, it is justified that the post ammonia annealing process is an effective way to fabricate TiN from TiO_2_.

### 2.5 Tungsten Nitrides

Tungsten nitride is a brown‐colored ceramic material that is electrically conductive and unstable in water. It is less commonly used than titanium nitrides. Tungsten nitride is extremely hard to synthesize in the past, as the incorporation of nitrogen atoms into the tungsten lattice is thermodynamically adverse at atmospheric pressure.^163^ Similar to the titanium nitride, ammonia reduction process^164^, ^165^ is utilized to produce tungsten nitride with the precursor of tungsten oxides and sulfides at a high temperature 600–900 °C. Additionally, CVD methods such as low pressure CVD,^78^, ^166^, ^167^ plasma enhanced CVD,^168^, ^169^, ^170^ and ALD^77^, ^171^, ^172^ have also been applied for the synthesis of tungsten nitride films and in the most cases, the stable β‐W_2_N phase is formed.^173^ In order to improve the surface area of tungsten nitride, Chakrapani et al.^174^ fabricated tungsten nitride nanowires on FTO substrate by a specially designed hot wire CVD method. The samples showed better electrocatalytic activity toward the HER than WO_3_ nano­wires, suggesting WN a promising catalyst material. Yu et al.^79^ synthesized holey tungsten oxynitride nanowires grown on carbon cloth via a two‐step process as supercapacitors electrode. Hydrothermal‐synthesized WO_3_ nanowires on carbon cloth were applied as the precursor and then followed by an ammonia reduction process for the conversion from WO_3_ to WON. During the annealing process, the smooth WO_3_ nanowires turned into rough ones with polycrystalline nature, and mesopores with size of 1–5 nm were formed on the nanowire, which would provide efficient channels, allowing fast and easy access of ions to the surface of electrode.

### 2.6 Molybdenum Nitrides

Molybdenum nitrides based materials have captured considerable attention since they displayed a series of unique and superior catalytic properties for hydrotreating^175^ and electrochemical catalysis.^176^, ^177^ Preparation methods of nanostructured molybdenum nitrides with high surface area have been reported using a variety of methods, but the majority of them are based on the ammonia reduction of Mo‐containing precursors. For example, in ref. 178 high surface area Mo_2_N (116 m^2^ g^−1^) have been prepared through nitridation of MgMoO_4_ in the flow of N_2_–H_2_ mixture at 800 °C.^179^ Xu et al.^180^ developed a self‐assembly method based on TPR technique to produce MoN hierarchical nanochex composed of single‐crystal nanowires. This growing process is realized with the help of a new interesting self‐assembly route, while the growth of MoN is limited in compressed hexagonal morphology.

### 2.7 Vanadium Nitrides (VNs)

VN is an active catalytic material with similar catalytic properties to noble metals. The traditional synthesis method of VN is called combustion synthesis. The vanadium powders react with nitrogen in very high pressure (>1 MPa) to form VN powder. The combustion‐synthesized VN samples are microscale sized and show dense structure. This method is not suitable for the synthesis of nanostructured vanadium nitrides. Currently, ammonia reduction of precursor with V sources is the dominant synthesis route for the VN nanostructures. Lu et al. used hydrothermal‐synthesized VOx nanowires as the precursor and followed by high‐temperature ammonia reduction to prepare highly porous VN nanowires. The above developed method is quite facile and high‐efficiency, and reproducible. 1D VN nanofibers are also synthesized via electrostatic spinning, which prepare precursors of polymer fibers, followed by high temperature heat treatment in ammonia.^181^ Zhou et al. synthesized VN powder by calcining V_2_O_5_ xerogel in an anhydrous NH_3_ ambient at 400 °C.^182^ Other two‐step ammonolysis methods followed heat treatment are also utilized to fabricate VN nanostructures. VCl_4_ and NH_3_ were adopted to prepare V(NH_3_)Cl precursor, which reacted with NH_4_Cl at high temperature to form VN products. The calculated crystalline size of VN is closely related to the heat treatment temperature.^66^

In addition to the ammonia reduction, sputtering is another popular method for the fabrication of VN films. VN thin films can be directly deposited on the substrate via reaction sputtering method from metal V target.^65^ The as‐deposited VN film has smooth surface without any grain boundary. Researchers also synthesized VN nanomaterials with various nanostructured morphologies. Mesocrystal nanosheets (MCNSs) of VN were prepared through a confined‐growth method from thermally stable layered vanadium bronze.^67^ The Na_2_V_6_O_16_ precursor nanosheets were synthesized via a hydrothermal method and they were subsequently annealed in an NH_3_ atmosphere at different temperatures. As for composites, VN/nitrogen‐doped graphene nanosheet composites as well as the VN/carbon nanotube was prepared by a facile sol–gel method followed by ammonia annealing.^68^, ^183^ And TiN/VN core–shell nanofibers were fabricated by the coaxial electrospinning plus post annealing in ammonia.^184^

### 2.8 Binary Metal Carbides/Nitrides

Previous research has demonstrated that some single‐phase carbides/nitrides are not electrochemically stable under catalytic/electrochemical condition in either acid or alkaline media.^104^ Numerous efforts have been made to improve the stability, including varying the composition and adding providing high surface area matrixes (e.g., graphene, CNTs, and carbon nanosheets) for them. One simple strategy is incorporating second metal to form bimetallic carbide, which enables tuning of the electronic properties of the mono‐phase material.^24^ This has been demonstrated by Navarro‐Flores et al.,^185^ who proposed a synergetic effect to account for the enhanced kinetics in hydrogen evolution for NiMo, NiW, and NiFe bimetallic carbide alloys.

Nevertheless, in most cases, the materials synthesized by conventional high temperature methods tend to form two separated monometallic carbides, or metal particles supported on the other carbide crystals, instead of bimetallic carbide alloys by lattice incorporation.^186^, ^187^ Michalsky et al.^188^ reported a CoWC material which contained two differences phases with 10% metallic Co and 90% WC, and exhibited a poor electrochemical stability. But this cannot be identified as bimetallic carbide. Ma et al.^189^ employed a lower temperature to produce a nanocomposite of cobalt molybdenum bimetallic carbides (Co_6_Mo_6_C_2_) supported on graphitized carbon by an ion‐exchange process followed by heat treatment. The obtained Co_6_Mo_6_C_2_ nanoparticles were well‐dispersed in the composite, and the graphitized carbons with a cage structure were interconnected with each other, which is beneficial to enhancing the stability and the conductivity of the carbides. Meanwhile, Liu et al.^60^ prepared carbon‐protected cobalt tungsten carbide at an even lower temperature. In particular, the sample was synthesized by a one‐step thermal treatment of a mixture of dicyandiamide, Co(NO_3_)_2_·6H_2_O and ammonium tungstate hydrate at 700 °C under nitrogen atmosphere. The as‐fabricated cobalt tungsten carbide particles were uniformly embedded in the carbon matrix and have average size of 5–20 nm. Very recently, a general method has been developed by Regmi and Leonard for transition metal (Ni, Co, Fe)‐incorporated bimetallic carbides of molybdenum and tungsten.^186^ Bimetallic carbides are synthesized by carbon thermal reduction of individual metal oxides with decolorizing carbon, and the process is based on reaction as follows (cobalt molybdenum carbide as an example)

(2.16) $$6{\mathrm{MoO}}_{3}+2{\mathrm{Co}}_{3}O_{4}+14C\;\;\to \;\;{\mathrm{Co}}_{6}{\mathrm{Mo}}_{6}C+13\mathrm{CO}\;(950{\;}^{}{}^{\circ}C)$$

The reaction temperature is an important factor for the formation of bimetallic carbides. The low carbon content phase Co_6_Mo_6_C will completely transform into a high carbon content phase Co_3_Mo_3_C when the reaction temperature is increased from 950 to 1010 °C, and similar for nickel and iron based bimetallic carbides, but only a slight enlargement of particles size is observed. Chen et al.^190^ reported the synthesis of NiMo alloy nitride nanosheets by reducing the mixture of ammonium molybdate and nickel nitrate in H_2_ at 400 °C, followed by nitridization in NH_3_ at 700 °C. The as‐prepared samples were found to contain a majority of γ‐Mo_2_N and Ni_2_Mo_3_N phases and exhibited an excellent HER activity.

## 3 Metal Carbides and Nitrides for Energy Storage Application

### 3.1 Li Ion Batteries

There are continuous efforts in the pursuit of high‐performance LIBs with high energy/power density to meet the increasing demand of modern electronics and transportation. Nowadays, commercial LIBs use graphite as the anode, in which Li ions intercalate between the graphite layers to form Li*_x_*C_6_ (*x* ≤ 1).^191^ However, graphite anodes have a rather low theoretical specific capacity of 372 mAh g^−1^.^192^ Generally speaking, there are three types of Li^+^ storage mechanisms.^193^ The first one is insertion of Li^+^ into existing vacancies in anode materials (such as graphite and TiO_2_). The main challenge is that most of the vacancies are not available for reversible storage of Li^+^ therefore the capacities are low. The second mechanism is alloying and de‐alloying of lithium with anode materials (such as Si, Ge, and Sn). This mechanism is associated with large (>200%) volume changes during the alloying and de‐alloying process which will disintegrate their intrinsic structure and result in poor cycling stability. The third mechanism of Li^+^ storage is based on conversion reaction between anode materials and Li^+^. This mechanism provides even higher energy densities. Most metals compounds (oxides, sulfides, phosphides, and alloys) fall in this category. But the associated volume changes, uncontrolled SEI film formation, and low conductivity result in poor rate performance, which are major obstacles in their commercialization.^194^, ^195^, ^196^ Among the explored anode materials for LIBs, TMCs and TMNs are expected to be attractive candidates as next‐generation anode materials for LIBs,^37^ due to the offered possibilities of a combination of different Li^+^ storage mechanisms. Similar to graphene, the MXenes with layered structure are able to store Li^+^ through insertion. Meanwhile, the existence of ionic and metallic bonds reveals the feasibility of alloying and conversion reaction with Li^+^ in TMCs and TMNs. Additionally, the excellent mechanical strength and hardness make them highly resistant to the deformation during the reaction. Research on TMNs and TMNs for LIBs is emerging and may bring up new opportunities by appropriate tuning the composition, morphology, doping, and hybridization with other materials.

#### 3.1.1 Titanium Carbides for LIB Applications

In the family of TMCs, titanium carbide is the most studied material as anode for LIBs. Zhou et al. have invested the lithium storage capability of Ti_3_C_2_ by DFT computations.^37^ The calculation results showed that Ti_3_C_2_ was a promising anode material for LIBs because of its outstanding properties, including high theoretical Li storage capacity, fast Li diffusion, positive electronic conductivity, and low operating voltage. This discovery becomes a milestone of the further understanding/development of TMCs for LIBs. Meanwhile, intensive efforts are being devoted to the energy storage application of the “MXenes”—2D carbide/nitride materials family including Ti_2_C, V_2_C, Nb_2_C, Ti_3_C_2_, Ta_4_C_3_, and Ti_3_CN. It is indicated that MXenes with *n* = 1, viz., M_2_X, which has less atomic layers compared with their higher order counterparts, M_3_X_2_ or M_4_X_3_, have higher gravimetric capacities.^197^ Several related works^38^, ^40^ revealed that functionalized MXenes are good electrical conductors, among which most are basically metals with rare exception, e.g., Ti_2_CO_2_ (where O is oxygen‐containing surface groups after HF treatment) is a semiconductor with a calculated band gap of 0.88 eV.^197^, ^198^ Naguib et al.^40^ studied the structure of MXene anode materials using ex situ XRD and showed that the Li ion storage mechanism was not a conversion reaction but intercalation of Li ions between the MXene layers. Moreover, according to the cyclic voltammetry analysis of Ti_2_C, they observed broad reversible peaks at 1.6 and 2.0 V versus Li^+^/Li during lithiation and delithiation process, respectively. These potentials are very close to those for TiO_2_ and Li*_x_*TiO_2_,^199^ so these peaks can be assigned to the following redox reaction

(3.1) $${\mathrm{Ti}}_{2}{\mathrm{CO}}_{x}+y{\mathrm{Li}}^{+}+ye^{-}\;\;↔\;\;{\mathrm{Li}}_{y}{\mathrm{Ti}}_{2}{\mathrm{CO}}_{x}$$

This layered material exhibited a stable capacity of 225 mAh g^−1^ at a C/25 rate and 110 mAh g^−1^ at a 1 C rate, which were lower than graphite and metal oxides. Nb_2_C and V_2_C have been prepared later with the same method, and demonstrated with a larger reversible capacity of 170 and 260 mAh g^−1^ at 1 C. Both of them showed excellent high cycling rate (10 C) capability. Modification of the surface structure of MXenes was conducted to further improve the performance.^200^ It is also^198^ reported that intercalation of organic molecules, dimethyl sulfoxide (DMSO), between Ti_3_C_2_ layers can delaminate the original structure and form nanosheets. This is beneficial to the Li^+^ diffusion and significantly increases the amount of Li adsorption. Anode made of the obtained Ti_3_C_2_ nanosheets showed a capacity of 410 mAh g^−1^ at 1 C, higher than graphite, and impressive high‐rate cycling capacity of 110 mAh g^−1^ at 36 C is achieved.

**Figure 8:**
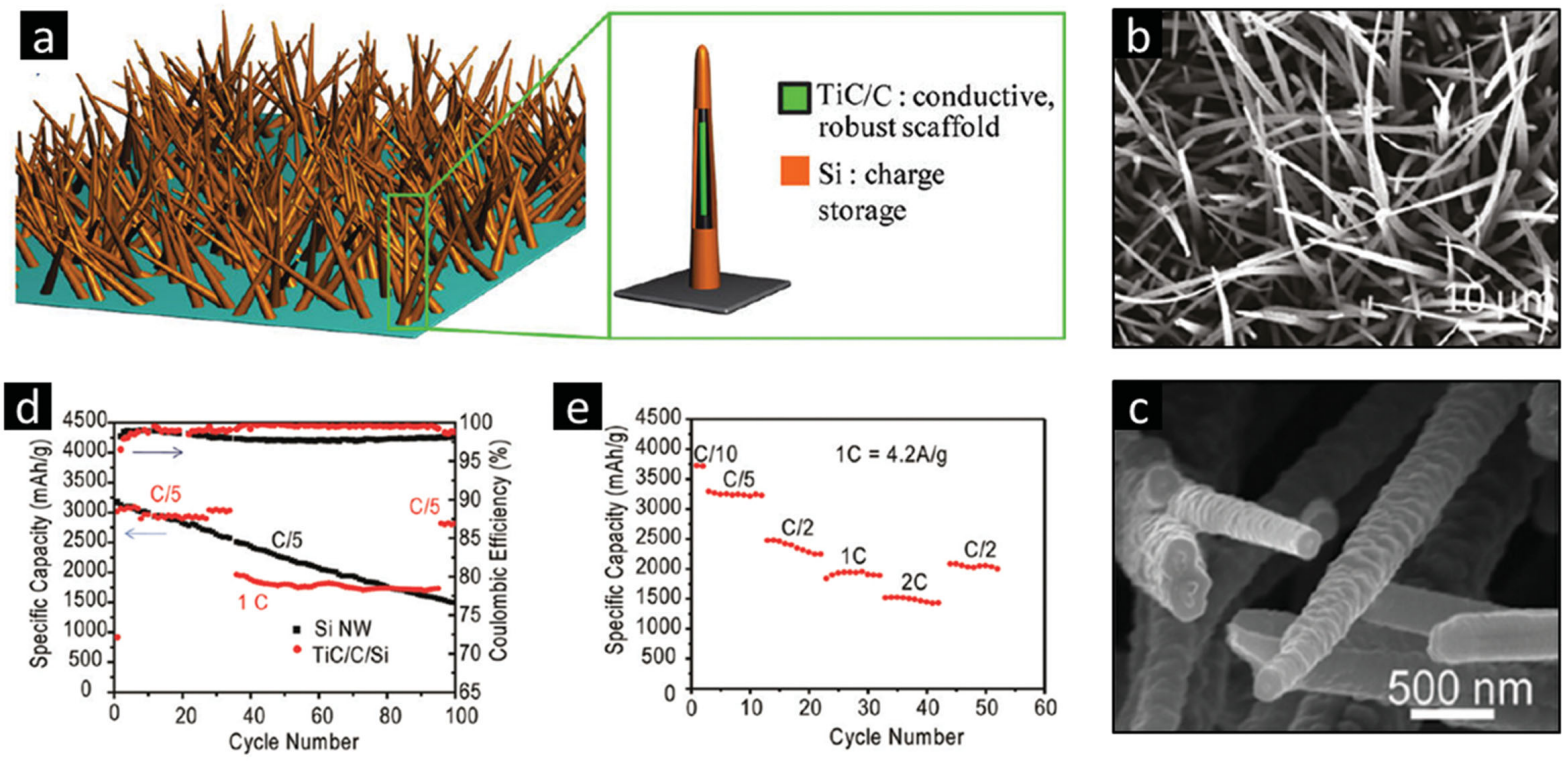
TiC/C/Si core/shell nanowires for LIBs anode. a) Schematics of the composite nanowire structure. TiC/C is inactive nanofiber scaffold and the amorphous Si is the active anode material. b,c) SEM images of TiC/C/Si nanocomposite nanowire electrodes. d) Comparison between the TiC/C/Si core/shell nanowires and pure Si nanowires in discharge specific capacity within 100 cycles at different C rates. e) Rate performance of the core/shell nanowire electrode (1 C = 4.2 A g^−1^). Reproduced with permission.^29^ Copyright 2011, American Chemical Society.

In addition, composition of the MXenes with other anode materials is proven beneficial to protecting the structure and enhancing conductivity. The most extensive research in this regard was conducted by Huang et al.,^46^ who synthesized a series of TMC nanowires (TiC, NbC, TaC, SiC, B_4_C, etc.) and designed a novel TiC/NiO core/shell composites. As the anode material for LIBs, the core/shell composite nanowire shows better cycling stability with a negligible capacity drop from 568.1 to 507.5 mAh g^−1^ during 60 cycles (90% capacity retention) and better rate performance of 367.6 mAh g^−1^ at a high rate of 3000 mA g^−1^ than the bare TiC or NiO electrode. It was also shown by the same authors that the TiC electrode can also exhibit evident double‐layer capacitive behavior, which is confirmed by both CV and EIS measurements. The Cui group^29^ fabricated TiC/C/Si nanocomposite nanofiber anode for LIBs (**Figure** **8**), which demonstrated a high specific capacity of 2800 mAh g^−1^ and a rather small capacity degradation (only 0.08% cycle^−1^) after 100 cycles. Here, we report a core–shell TiC/C/Si inactive/active nanocomposite for Si anodes demonstrating high specific capacity and excellent electrochemical cycling. The amorphous silicon layer serves as the active material to store Li^+^, while the inactive TiC/C nanofibers act as a conductive and mechanically robust scaffold for electron transport during the Li–Si alloying process. The core/shell architecture was also made to TiC nanoparticle core with polypyrrole (PPy) shell.^50^ The TiC@PPy LIB anode has a low specific capacity of only 110 mAh g^−1^, but the rate performance is quite impressive: 65% capacity retention at 20 C and nearly 40% at 400 C (equivalent to 40 A g^−1^). This indicates the advantage of the core/shell structure, in which the highly conductive TiC core and the thin PPy shell allow a fast diffusion of the electrolyte ions and efficient electron transfer.

#### 3.1.2 Other Transition Metal Carbides/Nitrides for LIB Applications

There are only a few investigations on the other TMCs and TMNs for LIBs until recent times. Several nanostructured TMCs including MoC_0.654_, WC, TaC, and NbC composited with vertically aligned carbon nanosheet and were proven with great lithium storage properties by a simple salt temple method. According to the representative cyclic voltammogram obtained, they indicated that the mechanism of lithium storage in metal carbides was the conversion and alloying reactions between Li^+^ and the metal phase as follows (e.g., molybdenum carbide)

(3.2) $$y{\mathrm{Li}}^{+}+{\mathrm{Mo}}_{0.654}C+ye^{-}\;\;\to \;\;\mathrm{Mo}+{\mathrm{Li}}_{y}C$$

Simultaneously, no evident platform is observed throughout the whole cycle, which could be due to the comparatively disordered structure of the carbon nanosheets. This Mo_0.654_C@CNS sample showed an initial coulombic efficiency of 68% in which the capacity loss was due to the formation of SEI layer. An average capacity as high as 1010 mAh g^−1^ was retained at a current density of 200 mA g^−1^. It is conclusive that MoC@CNS composites are good anode materials for LIBs.

VN is also considered as an alternative anode material for LIBs because of its merits such as high corrosion and temperature resistance, and chemical stability.^15^ Several reports have showed that VN displays a larger capacity for thin film lithium battery than other TMNs.^201^, ^202^ For instance, Sun and Fu^65^ developed a VN thin film exhibiting a very high discharge capacity of 1500 mAh g^−1^. However, despite good electronic conductivity, VN nanoparticles were reported to show a narrow rate capability,^203^ which could be possibly related to the formation of metallic M (or MN_1−*x*_) and Li_3_N phase. This reversible conversion reaction can be represented as follows^204^

(3.3) $$x\mathrm{MN}+3x{\mathrm{Li}}^{+}+3xe^{-}\;\;↔\;\;xM+x{\mathrm{Li}}_{3}N\;(M=V,\mathrm{Ti},\mathrm{etc}.)$$

Because of this conversion reaction, the diffusion of ions suffers from a relatively slow kinetics. Furthermore, smaller and thinner nanoparticles are formed by Li ions incorporation into the lattice, leading to disintegration of the nanostructures, which is another obstacle for the fast lithium‐ion migration rate inside the particles. This issue can be circumvented by embedding VN particles on highly conductive scaffold such as nitrogen‐doped graphene nanosheets.^183^ It is suggested that the enhancement of rate performance originates from a synergistic effect between VN and graphene. The graphene network helps to buffer the structural changes and the spacing between the layers of graphene may also provide extra active sites for lithium ions intercalation. The enhanced transfer of ions/electrons can improve the reaction kinetics leading to better rate capability. Additionally, bimetallic nitride (Ti and V) was also developed with an attractive electrochemical performance of about 650 mAh g^−1^,^205^ higher than that of TiN/C composites (200 mAh g^−1^).

### 3.2 Sodium Ion Batteries

**Figure 9:**
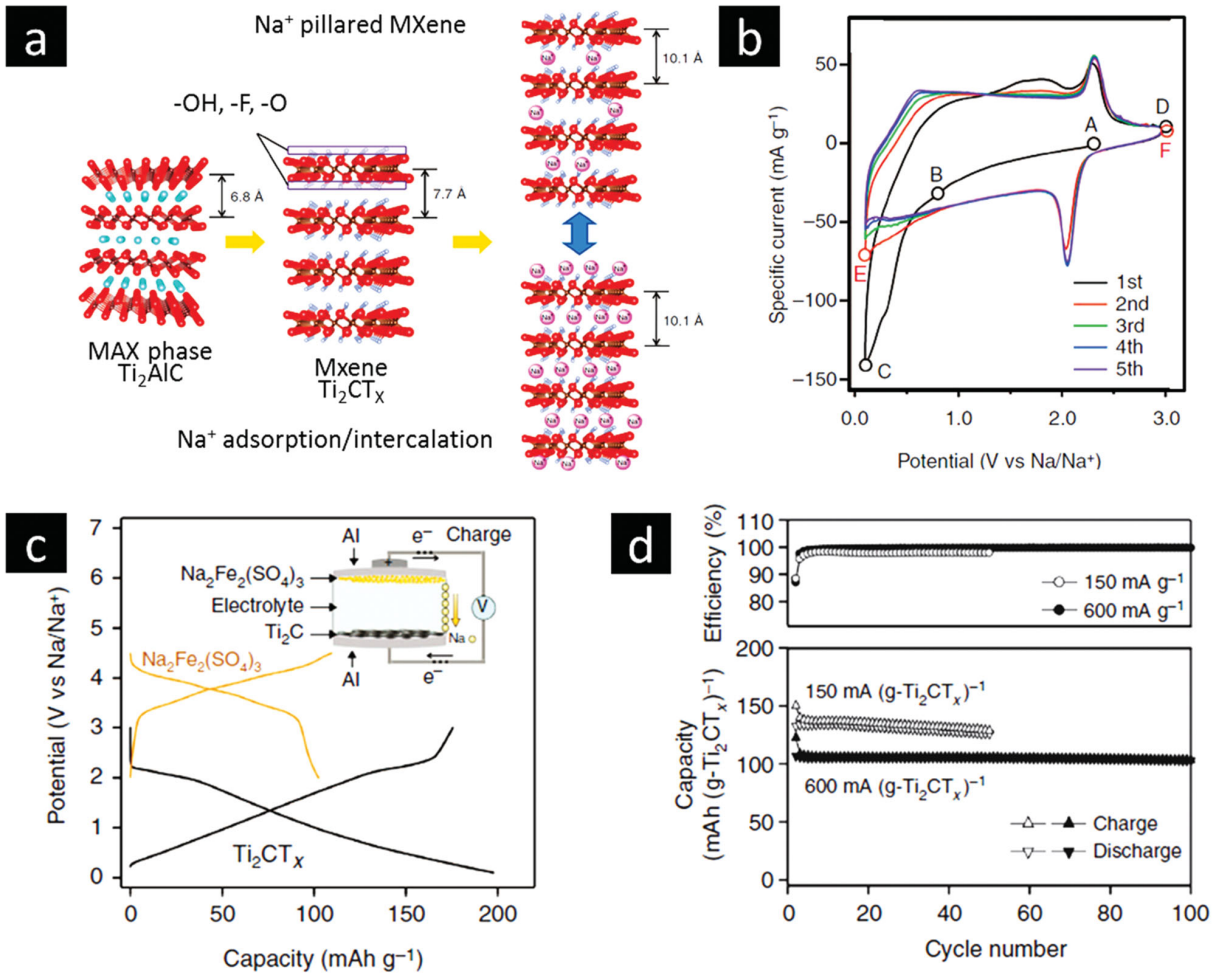
Mxene Ti_2_CT*_x_* as the anode material for Na‐ion storage. a) Schematics of the reaction mechanism of Ti_2_CT*_x_* by electrochemical activation. b) CV curves in first five cycles in a 1 m NaPF_6_/EC‐DEC electrolyte. Scan rate: 0.2 mV s^−1^. c) Charge/discharge curves of a full cell containing Ti_2_CT*_x_* and Na_2_Fe_2_(SO_4_)_3_ electrodes. d) Cycle performance of the full cell at two current densities (cutoff voltages: 0.1–3.8 V). Reproduced with permission.^213^ Copyright 2015, Nature Publishing Group.

Despite the ubiquitous usage of lithium batteries, research on new‐generation batteries is booming. Rechargeable sodium ion batteries are regarded as future cheaper power source than LIB (Na is fourth most abundant element in the Earth crust).^7^, ^206^, ^207^ In contrast to LIBs, graphite does not intercalate sodium to any appreciable extent and is electrochemically irreversible. Many other anode materials have been demonstrated for Na insertion, such as carbonaceous,^208^ metal oxide,^209^ and phosphorus^210^ materials. To date, relatively few reports are available on TMCs or TMNs for SIBs, until recently the 2D TMCs, MXenes, were reported to be a promising anode material for SIBs (as well as multivalent metals).^211^ DFT calculations by Yang et al.^212^ indicated that, from the viewpoint of voltage, Ti, V, Cr, Mn, and Mo carbides are suitable materials for SIB anodes, and they are able to cycle at high rates because of low Na ion migration barriers. Wang et al.^213^ have investigated the performance of activated MXene Ti_2_C nanosheets (see **Figure** **9**). The electrode delivered a high specific energy of 260 Wh kg^−1^ at a high specific power of 1.4 kW kg^−1^ and good stability, demonstrating the promising high‐power performance of the cell in Na‐ion storage. Furthermore, according to Xie et al.,^214^ the OH‐terminated MXenes may have multistage Na ion storage mechanisms: first, the OH‐terminated MXenes decompose into bare MXenes and metal oxides when in contact with multivalent metals; the sodium ions will be stored in the form of sodium oxides first and next can intercalate into bare MXenes to form the first metal layer. The authors also suggested that bare MXene nanosheets have higher capacity for metal ion storage, but with drawback of instability due to their more active surface. Finally, vanadium carbide based MXene has been tested as positive electrode material for SIBs, although a low capacity of 50 mAh g^−1^ with a maximum cell voltage of 3.5 V was shown.^215^ Overall, in the present stage, the SIB performance of TMCs or TMNs electrode is still far from being satisfactory, and more investigation is required to achieve high capacity and long‐cycle stability.

### 3.3 Supercapacitors

SCs, also called electrochemical capacitors, are regarded one type of very promising energy storage technology since they possess higher power densities than batteries while their energy densities are superior to conventional capacitors. Obviously, SCs could play a role to bridge the energy and power gap between conventional capacitors and batteries.^216^ Based on the energy storage mechanism, SCs can be classified into two main categories: electrochemical double layer capacitors (EDLCs) and pseudocapacitors. The energy storage mechanism of EDLCs is physical charge accumulation at the interface between electrolyte and electrode without involving change in volume lattice.^217^ It is a non‐faradaic process because there is no charge transferring across the interface. Consequently, the cycling life of EDLCs is long, typically 10^5^ cycles.

Different from EDLCs, the pseudocapacitors involve surface redox reactions. With a potential applied to a pseudocapacitor, fast and reversible faradaic redox reactions will take place on the available surfaces of electrode materials and accompanied by charge transfer. The faradaic process includes reversible absorption, surface redox reactions in transition metal oxides, and reversible electrochemical doping–undoping process in conducting polymers. In general, the pseudocapacitors exhibit higher capacitance than EDLCs since the faradaic reactions can take place both on the surface of electrodes and in the interior of them, especially for ultrathin nano‐sized materials.^218^, ^219^ However, the pseudocapacitors often suffer from inferior rate capability and unsatisfactory cycling life compared with EDLCs.

In recent years, transition metal nitrides and carbides have been demonstrated as active materials for SCs due to their high electrical conductivity and reactivity.^220^, ^221^, ^222^ Noticeably, enhanced high‐rate capability and excellent cycling life have been achieved in these systems, as compared to the commonly researched metal oxides.

#### 3.3.1 Vanadium Nitride for SC Applications

Among transition metal nitrides and carbides, vanadium mono­nitride has been the most well‐studied material for SCs due to its high specific capacitance (the highest reported specific capacitance of VN is 1340 F g^−1^)^66^ and good electrical conductivity (1.6 × 10^6^ S m^−1^). Choi et al. synthesized nanocrystalline VN through a two‐step ammonolysis reaction of VCl_4_ in anhydrous chloroform at a low temperature.^66^ The obtained VN had small crystallite size of 6.33 nm, resulting in a high specific surface area of 38.8 m^2^ g^−1^. As a result, it exhibited a fairly high specific capacitance of 1340 F g^−1^ in 1 m KOH electrolyte. They found that the electrochemical performance of VN was closely related to the temperature of heat treatment. When the temperature increased to 1000 °C, the crystallite size increased to 58 nm and the specific surface area decreased to only 2.4 m^2^ g^−1^, leading to a low specific capacitance of 58.3 F g^−1^. It should be mentioned that most of the TMNs (such as VN and TiN) are used as anode of supercapacitors, so the electrochemical properties of their single electrodes are usually tested in negative potential windows. The origin of the capacitance of most TMNs is essentially the surface‐modified oxides or oxynitrides as well as double‐layer charging. It is speculated that the capacitance of the nanostructured VN electrode originates from a combination of EDLC and pseudocapacitance. The possible reversible redox reations in a LiCl electrolyte can be described as follows

(3.4) $$\begin{array}{l} V_{x+2y/3}N_{x}O_{y}+zM^{+}+ze^{-}\;\;↔\;\;V_{x+(2y-z)/3}N_{x}O_{y}M_{z}^{z+}\\ \;\times (M^{+}={\mathrm{Li}}^{+},H^{+}\ldots ) \end{array}$$

Kumta's group reported nanocrystalline VN synthesized via a simple two procedure involving milling induced mechano‐chemical reaction of V_2_O_3_ and Li_3_N mixture.^223^ However, the specific capacitance was only in the range of 25–60 F g^−1^ as a result of the relatively larger particle size and lower surface area.^66^ Since VN nanoparticles usually suffer from poor electrochemical performance due to its aggregation and ineffective contact, 1D VN nanofibers were synthesized through electrostatic spinning followed by high temperature heat treatment in ammonia.^181^ The cross‐linked VN nanofibers composed of nanoparticles could accelerate the transport for ion and charge. Owing to the unique nanoarchitecture, VN nanofibers exhibited a high specific capacitance of 291.5 F g^−1^ at 0.5 A g^−1^. Bi et al. synthesized MCNS of VN through a confined‐growth route from thermally stable layered vanadium bronze.^67^ This kind of material exhibited a high electrical conductivity of 1.44 × 10^5^ S m^−1^ at room temperature due to their single‐crystalline like long‐range electronic connectivity. Its specific capacitance was about 1937 mF cm^−3^. Despite these encouraging results, however, the vanadium nitrides usually display a limited rate capability. To overcome this problem, Ghimbeu et al.^68^ have fabricated a VN/CNT composite, which effectively utilized the high conductivity, high surface area, and unique mesoporous network of CNT. This composite exhibited a high capacitance retention (58% relative to the specific capacitance at 50 mA g^−1^) at a high current density (30 A g^−1^) compared with pristine VN (7%). The VN nanoparticles embedded within an N‐doped carbon matrix, with tunable pore size and nanoparticle size, and surface area, also exhibited a good rate capability.^224^ In addition, VN can also be used for flexible SCs electrode. Lu et al.^34^ fabricated a VO*_x_*//VN quasi‐solid‐state asymmetric supercapacitor, which provided a stable electrochemical window of 1.8 V and excellent cycle stability with 12.5% loss of capacitance after 10 000 cycles. It exhibited a high energy density of 0.61 mWh cm^−3^ at 0.5 mA cm^−2^ with a high power density of 0.85 W cm^−3^ at 5 mA cm^−2^.

#### 3.3.2 Titanium Nitride for SC Applications

Compared to VN, TiN generally has a higher electronic conductivity but the capacity is relatively low. With the help of ALD and post ammonia reduction, we prepared TiN on vertical graphene nanosheets (TIN/GNS) composites. The as‐prepared TiN had pore size varied from 3 to 10 nm and showed highly porous structure. Coupled with the Fe_2_N/GNs, full cells were constructed and showed good flexibility, high‐rate capability, and 98% capacitance retention after 20 000 cycles.

**Figure 10:**
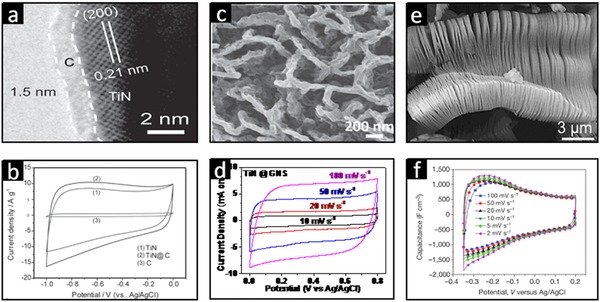
Nanostructured titanium nitride and carbides for supercapacitors. a) HRTEM images of TiN@C core/shell nanowires and b) the CV curve collected at a scan rate of 100 mV s^−1^ in comparison to pristine C and TiN electrodes. c) SEM image of TiN nanoparticles supported on graphene nanosheets (GNS) and d) the corresponding CV curves of the TiN@GNS electrode tested in 1 m LiCl electrolyte. e) SEM image of the2D layered Ti_3_AlC_2_ and f) the CV profiles at different scan rates for a 5 μm thick electrode in 1 m H_2_SO_4_. a,b) Reproduced with permission.^153^ Copyright American Chemical Society. c,d) Reproduced with permission.^76^ e,f) Reproduced with permission.^31^ Copyright 2014, Nature Publishing Group.

Alternatively, incorporating TiN and VN into an electron and ion mixed transport nanocomposites is expected to deliver excellent electrochemical performance for energy storage. Simply by electrospinning and post annealing in ammonia, coaxial TiN and VN core–shell nanofibers were synthesized for supercapacitor electrode materials.^184^ This composite fibers make advantages of both TiN and VN, which delivered a higher specific capacitance (247.5 F g^−1^ at 2 mV s^−1^) and better rate capability (65% capacitance retention at 50 mV s^−1^) than pristine nitride nanofibers. Grote et al.^155^ synthesized self‐supported TiN nanotube arrays coated with carbon with the aid of anodic aluminum oxide templates and ALD technique. Currently, TiN is found to be easily oxidized in aqueous solutions. So a key challenge for metal nitride materials in supercapacitor application is to prolong its cycle life. A carbon coating layer with optimal thickness is always helpful in maintaining the long‐term cycling stability, and also inhibiting the electrochemical oxidation of metal nitrides during cycling. This result is supported by the research by Lu et al., in which an ultrathin and stable amorphous carbon protective coating was applied to TiN nanowires by the hydrothermal decomposition of glucose (**Figure** **10**a,b).^153^ The modified TiN@C electrode achieved a specific capacitance of 124.5 F g^−1^ at 5 A g^−1^ compared to higher than the TiN (107 F g^−1^). The slight increase in capacitance probably originates from the double‐layer capacitance of the carbon shell. With the carbon coating, the electrode retained 91.3% capacitance after 15 000 cycles compared to 9.1% for the pristine TiN nanowires electrode. TiN nanoparticles anchored on graphene nanosheets by converting ALD‐coated TiO_2_ have also been applied as a stable supercapacitor electrode in nonaqueous LiCl electrolyte with stable capacitance up to 20 000 cycles (Figure 10c,d).^76^

#### 3.3.3 Other Transition Metal Carbides/Nitrides for SC Applications

Pande et al. synthesized nanostructured V, Mo, and W nitrides and carbides for supercapacitors in aqueous KOH and H_2_SO_4_ electrolytes.^225^ The obtained γ‐Mo_2_N and β‐W_2_C provided decent gravimetric capacitance or areal capacitance in KOH and/or H_2_SO_4_ electrolytes, but the electrochemical performance of VC and β‐W_2_N was not very good. More recently, the MXene family is the rising star for SCs application. 2D titanium carbide (Ti_3_C_2_, a member of the “MXene” family) is produced by etching aluminium from titanium aluminium carbide (Ti_3_AlC_2_, a “MAX” phase) in concentrated hydrofluoric acid. This kind of material is proven to have high electrochemical performance (see Figure 10e,f). The rolled films of conductive 2D titanium carbide “clay” reported by Gogotsi team^31^ provide a volumetric capacitance up to 900 F cm^−3^ or 245 F g^−1^. Delamination or intercalation surface treatments of 2D titanium carbides generated oxygen‐containing functional groups leading to increase in capacitance.^226^ For the cubic phase tungsten oxynitride, the nonmetal atoms (N, O) occupy the interstitial sites in the metal lattice; this unique crystal structure corresponds to high conductivity, thermal stability, and hardness. Holey tungsten oxynitride nanowires were fabricated by a two‐step process and used as anodes of asymmetric supercapacitors and microbial fuel cells, with high power densities up to 10 kW kg^−1^.^79^

## 4 Transition Metal Carbides and Nitrides Electrodes for Electrochemical Catalysis

Hydrogen has the highest specific energy density among any known fuel, and is reconcilable with most electrochemical processes due to its advantage of CO_2_‐free emissions.^5^, ^227^ To date, various types of electrochemical devices such as fuel cells and photo‐electrochemical cells^228^ are designed to utilize or produce hydrogen and oxygen for storing energy or generating electricity. These devices involve electrochemical reactions including HER, OER, and ORR,^228^, ^229^ for which high‐performance catalysts are required to improve the reaction efficiency. Unfortunately, expensive Pt‐group metals (Pt, Ru, Rh, Ir, and Pd) have been used predominantly for most of the aforementioned reactions. The high price and limited supplies of these precious metals greatly restrict the development and large‐scale production of the requiring devices. Many attempts have been taken to substantially reduce the loading of precious metals or alternatives to Pt‐based catalysts, and one of the most exciting discoveries is the Pt‐like catalytic behavior of WC in 1973 by Levy and Boudart.^12^ This discovery opened up new possibilities to investigating and designing the “Pt‐like” properties of catalysts based on tungsten carbide or other TMCs or TMNs.

**Figure 11:**
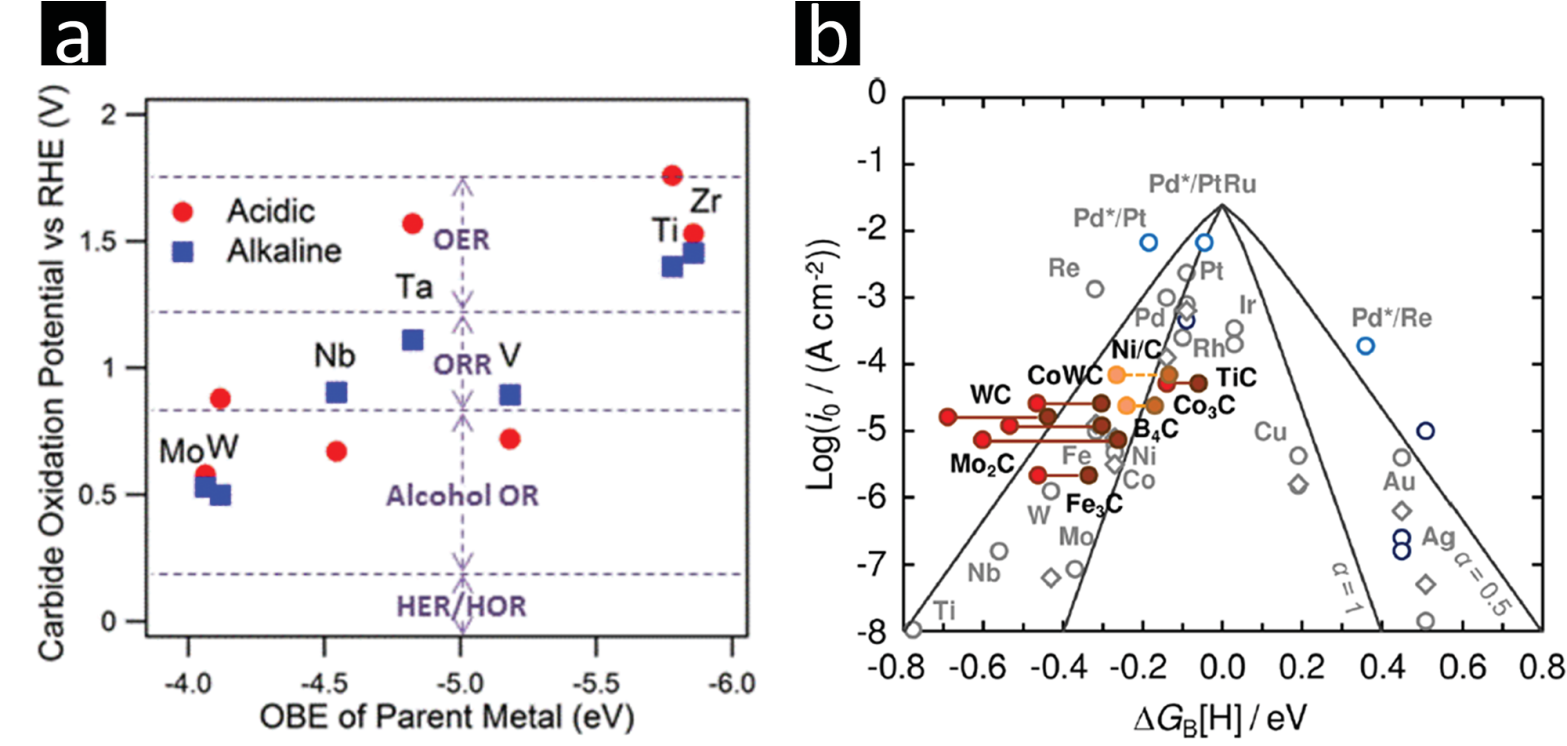
Calculated trends in metal carbide for electrochemical catalyst. a) Plot of the oxidation potential as a function of DFT‐calculated oxygen binding energy of the parent metal. b) Volcano plot between the exchange current density and the hydrogen binding energy. a) Reproduced with permission.^233^ Copyright 2014, American Chemical Society. b) Reproduced with permission.^188^ Copyright 2014, American Chemical Society.

Generally, several elementary processes will occur in the course of an overall electrochemical catalytic reaction, including adsorption and desorption of atoms/function groups, breaking and formation of bonds, and electron transfer.^24^ Calculations by DFT allow prediction of the electronic and chemical properties of metal‐modified carbide surfaces, as well as determination of the binding energies (e.g., hydrogen binding energy (HBE) and oxygen binding energy (OBE)) of reactant molecules.^230^, ^231^, ^232^ The binding energies provide information on electronic properties of selected surfaces, which is important to understanding the relationship between catalyst properties and catalytic activity. For example, Kimmel et al. investigated the oxidation potential as a function of DFT‐calculated OBE of the parent metal,^233^ and established the relevance between OBE and the onset of oxidation for the TMCs (**Figure** **11**a). A general trend in the reactivity of metal carbide surfaces has also been developed into the volcano plot between exchange current density and the HBE (see Figure 11b).^188^

### 4.1 Hydrogen Evolution Reaction

HER is of great importance for a variety of electrochemical processes, which provide an essential link between renewable energy sources and energy conversion applications such as reversible hydrogen fuel cell. The HER (2H^+^ + 2e^−^ → H_2_) requires efficient electrocatalysts to accelerate the decomposition of water. In the two‐electron‐reaction model, cathodic HER in acidic aqueous media occurs with a “discharge step” and an electrochemical desorption step. The first step is the Volmer reaction, during which an intermediate state of a hydrogen atom is generated by transfer of one electron to a proton at the catalyst surface

(4.1) $$H_{\left(\mathrm{aq}\right)}^{+}+e^{-}\;\to \;H_{\mathrm{ad}}$$

Subsequently, two possible routes for the HER will follow. One is the Heyrovsky's reaction

(4.2) $$H_{\mathrm{ad}}+H_{\left(\mathrm{aq}\right)}^{+}+e^{-}\;\;\to \;H_{2}\;(g)$$

The other is the Tafel recombination reaction

(4.3) $$H_{\mathrm{ad}}+H_{\mathrm{ad}}\;\;\to \;\;H_{2}\;(g)$$

It is generally considered that the Volmer reaction is rapid and so either of the rest two reactions (Tafel reaction and Heyrowsky reaction) becomes the rate‐limiting step. Tafel slope is often applied as an important factor to determine the dominant mechanism. In the Tafel plot, it is favorable to achieve a low Tafel slope at a large exchange current density. Therefore, an efficient HER electrocatalyst should be a material that can lower substantially the overpotential.

Platinum has been the most efficient HER catalyst with a low overpotential (0.02 V at 1 mA cm^−2^) in acidic electrolyte,^234^ but the obvious drawback is its high cost and low earth abundance. The mainstream of the current research is to develop noble‐metal‐free alternatives. Transition metal carbides have been extensively investigated for decades as the promising HER catalysts.^230^ It is worth noting that very few catalysts are active at both pH 0 and 14. Pt is one such catalyst, so as to tungsten carbide and molybdenum carbide. Incorporation of carbon into the early transition metals will result in an expansion of the lattice constant, and a broadening in the *d*‐band structure of the metals due to the hybridization between metal *d*‐orbitals and the carbon *s*‐ and *p*‐orbitals. This hybridization in turn gives rise to the catalytic properties of metal carbides resembling that of noble metals.^231^, ^233^, ^235^

#### 4.1.1 Tungsten Carbides for HER Application

**Figure 12:**
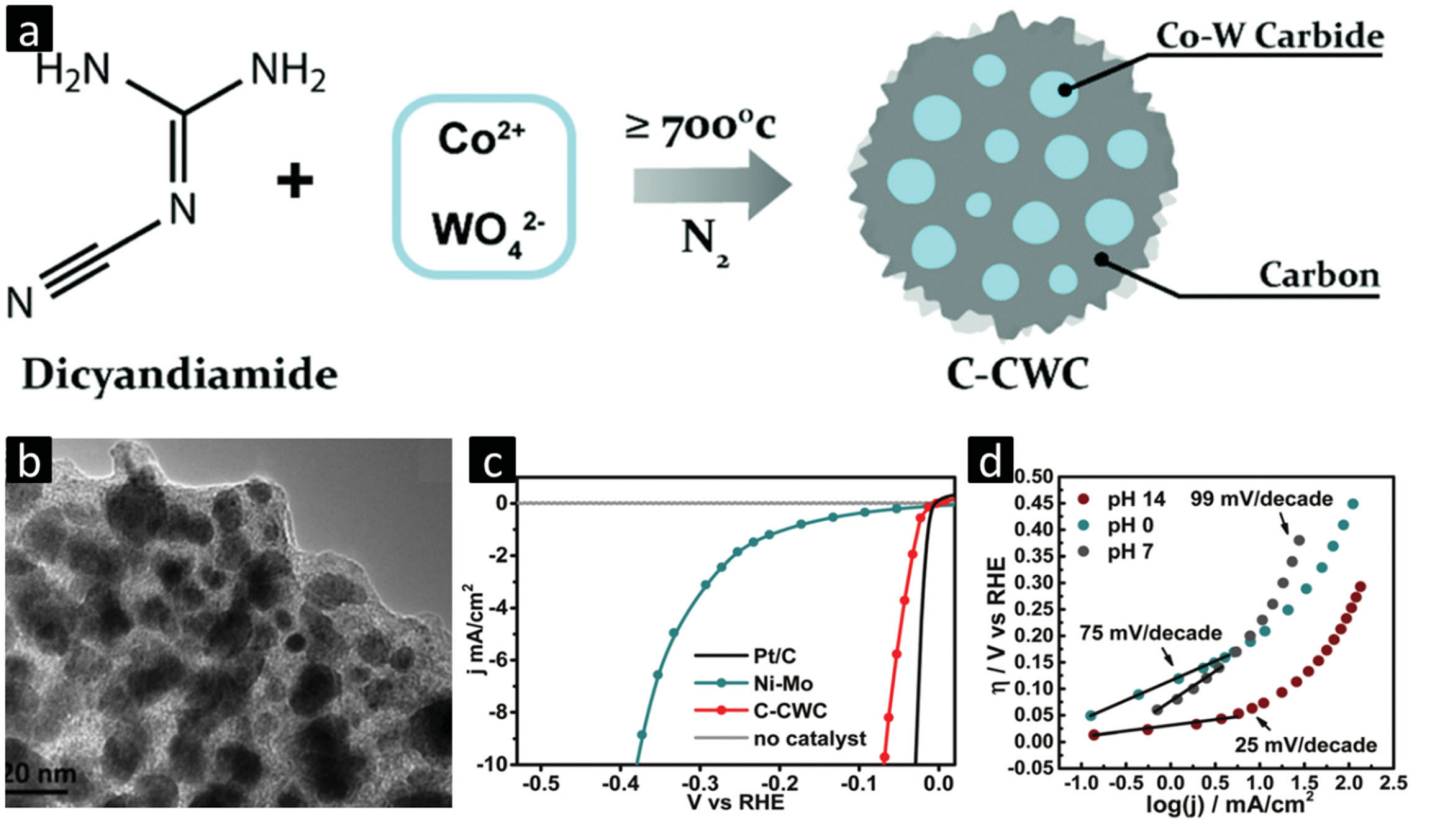
Hybrid electrocatalyst for HER in both basic and acidic electrolyte consisting of carbon and cobalt–tungsten carbide (CWC) nanoparticles. a) Schematics of the synthesis process. b) TEM image of C‐CWC. c) Comparison of the *J*–*V* curves of three types of electrocatalyst (5 wt% Pt/C, Ni–Mo alloy, and C‐CWC) and no catalyst in 1 m KOH solution. d) Tafel plot of the C‐CWC material in different solutions: 0.5 m H_2_SO_4_ (pH 0), phosphate buffer (pH 7), and 1 m KOH (pH 14). Reproduced with permission.^60^ Copyright 2015, Elsevier.

Tungsten carbide has been demonstrated as a highly active HER catalyst even as bulky particles.^236^ This is also supported by the results published by Lee et al.,^237^ who investigated several different IVB–VIB transition metal carbides and nitrides bulky particles originating from industrial production to act as HER electrocatalysts in 0.1 m H_2_SO_4_. The results showed that the onset potential *η*_onset_ of tungsten carbide for the HER was about 184 mV versus Ag/AgCl and the overpotential was 444 mV versus Ag/AgCl at a current density of 20 mA cm^−2^. Liu et al.^60^ synthesized a hybrid structure consisting of carbon and cobalt–tungsten carbide (CWC) particles that displayed efficient HER performance in alkaline media as shown in **Figure** **12**. The above catalyst afforded a current density of 10 mA cm^−2^ at a low overpotential of 73 mV, compared to 33 mV of Pt/C at the same current density. In addition, thermal annealing at or above 700 °C increased the bimetallic crystallinity of Co–W carbide phase and thus improved the catalytic activity. Yan and his co‐workers^61^ have reported an electrocatalysts of layered MoS_2_ supported on reduced graphene oxide decorated with tungsten carbide. The as‐prepared composite showed an onset potential of −0.11 V versus RHE in 0.5 m H_2_SO_4_ solution and the Tafel slope of ≈41 mV dec^−1^, which was comparable to a commercial Pt catalyst (≈30 mV dec^−1^). The above examples show that the ternary catalysts can have better catalytic activity than the individual constituents. Taking the Yan's work as example,^61^ the enhancement could be explained by a positive synergistic effect between WC and co‐catalysts. It is suggested the WC decorated RGO co‐catalyst provides a suitable substrate for the formation and stabilization of the layered MoS_2_ with increased abundance of exposed edges as the active catalytic sites for the HER. The composites also possess high conductive matrix providing better electron transfer path. In addition, somehow the combination of the three HER‐active components (MoS_2_, WC, and RGO) renders more catalytic sites and better anti‐poisoning, and lowers the energy barrier of HER. This synergistic effect has also been investigated by other groups.^36^, ^60^, ^185^, ^238^, ^239^, ^240^ Chen et al. found that growth of the W_2_C–WN nanoparticles onto graphene nanoplates would be more efficient in the HER. The W_2_C–WN@GnP had an overpotential of about 120 mV at the current density of 10 mA cm^−2^ with, while the bulk W_2_C catalyst required 336 mV at the same current density. Other authors^241^ have suggested that WN was stable in alkaline electrolytes for HER, but with a poor performance. It was also indicated that the proportion of W_2_C–WN in the tungsten phases had an important influence on the HER performance.

#### 4.1.2 Molybdenum Carbides for HER Application

Similar to tungsten carbides, molybdenum carbide has also been demonstrated an efficient HER catalyst. Vrubel and Hu have investigated the HER performance of commercially available Mo_2_C.^242^ It was shown that the activity of Mo_2_C in alkaline media is comparable to that in acid media: The overpotential for a current density of 10 mA cm^−2^ was 210 mV at pH 0 and 190 mV at pH 14. The particle size is found to be an important factor to catalytic properties. In addition to high surface areas, small‐sized particles correspond to short diffusion and transport pathways of electrolyte ions. Ma et al. have prepared α‐Mo_2_C nanoparticles (with grain size around 11 nm) via an urea glass route showing superior performance in both acid and alkaline electrolyte:^51^ The overpotential was up to 198 mV in 0.5 m H_2_SO_4_ and 176 mV in 1 m KOH, with a corresponding Tafel slope of 56 and 58 mV dec^−1^, respectively. In contrast, the commercial Mo_2_C catalyst (with the average grain size of 125 nm) exhibited a larger Tafel slope of 100 mV dec^−1^. This seemly size effect was consistent with the observation that the Tafel slope values increased with the size of the particles.^57^, ^242^

High surface areas are advantageous but not always the only reason for higher electrocatalytic activity than bulk counterparts. Designing nanostructure catalyst uniform dispersed in mesoporous matrix is also an effective strategy to lift the diffusion and transport kinetics. Wu et al.^53^ prepared porous nano‐octahedron samples by embedding η‐MoC*_x_* into an amorphous carbon matrix, which provided a 3D matrix for loading and stabilizing η‐MoC*_x_*. Such composite exhibited outstanding HER performance with onset potential of about 25 and 80 mV versus RHE in 0.5 m H_2_SO_4_ and 1 m KOH, respectively. Advantage over irregular MoC*_x_* nanoparticles was clearly demonstrated therein.^53^ In this structural design, the octahedral nanocrystallites were protected by the amorphous carbon matrix for superior robustness.

Similar structure design and performance improvement can also be realized by incorporating electrically conductive supports (e.g., carbon nanotubes, graphene, and porous current collectors). A series of TMCs including MoC_0.654_, WC, TaC, and NbC composited with vertically aligned CNS for HER have been investigated by Zhu et al.^33^ It was shown MoC0.654@CNS and WC@CNS showed relatively low overpotentials (220 mV at a current density of 10 mA cm^−2^) compared to pure Ni foam (320 mV) in alkaline medium. Also, there was no evident loss in current density for 5 h at a constant potential of −0.22 V versus RHE, to which an excellent electrical conductivity of the vertical‐aligned CNS and their strong interaction with the current collector certain play roles.

Taking the results above, it is concluded that the low‐cost carbide supported electrocatalysts are outstanding electrocatalysts for HER and have catalytic activity in both acid and alkali media. Composites with carbon supports can further enhance the performance of these carbides nanomaterials.

### 4.2 Oxygen Evolution Reaction

OER is another important half reaction for water splitting reaction. The mechanism for oxygen evolution in the acid media can be generally described by the following reactions^243^, ^244^

(4.4) $$A_{S}+H_{2}O\;\;\to \;\;A_{S}-{\mathrm{OH}}_{\mathrm{ads}}+H^{+}+e^{-}$$

(4.5) $$A_{S}-{\mathrm{OH}}_{\mathrm{ads}}\;\;\to \;\;A_{S}-O_{\mathrm{ads}}+H^{+}+e^{-}$$

(4.6) $$A_{S}-O_{\mathrm{ads}}\;\;\to \;\;A_{S}+1/2\;O_{2}$$

where A_S_ stands for active sites at the catalyst surface, and A_S_–OH_ads_, A_S_–O_ads_ are two adsorption intermediates. Compared to HER, OER is more complex because it involves a four‐electron oxidation process and two water molecules to form one O_2_ molecule.^245^ In this regard, good catalysts for OER is far more indispensable than for HER. Ru, Ir, and their oxides are common high‐performance catalysts for OER,^242^ but like Pt, their high‐cost and scarcity are the major hurdle to the large‐scale commercialization. In addition, an OER catalyst should have a good resistance to the oxidation because the formed oxygen‐containing species are a strong oxidizing agent.^246^ The absorbed species at the metal surface can obstruct the approach of oxygen‐containing species to the surface sites, and thus have strong influences on the overpotential of the oxygen anode operation.

#### 4.2.1 Transition Metal Carbides for OER

There are still very few publications in the field of TMCs/TMNs as the catalyst for OER. OER requires the catalyst to be immune to the oxidation of oxygen and stable in aqueous media. Unfortunately, most of TMNs are not stable during OER in aqueous media, but some TMCs are qualified. Esposito et al.^230^ investigated the correlation between the stability of the TMC thin films and the oxygen binding energy of the parent metal. Their results indicated that all metal carbides, especially for tantalum carbide and titanium carbide, showed good chemical stability for OER. Ignaszak et al.^104^ further improved the electrochemical stability of TiC under the operating conditions of fuel cells by forming TiC@TiO_2_ core–shell composite. Another group^247^ improved the durability by mixing the TiC with multiwalled carbon nanotubes (MWCNTs). Another functionality of metal carbides in OER is to act as support for active catalytic materials. In the work of Ma et al.,^246^ an Ir/TiC catalyst was developed by depositing Ir nanoparticles on the surface of TiC support. It was found that the peak current density at 1.5 V on the Ir/TiC was about nine times higher than those of unmodified Ir catalyst. Polonský et al.^248^ reported that TaC could act as a stable strong backbone for IrO_2_ electrocatalyst in the anodic OER. It was demonstrated that the electrocatalysts with IrO_2_ of 50 wt% showed OER properties similar to those of pure IrO_2_. Based on that work, Nikiforova et al.^249^ continued to evaluate TaC and NbC as supports for the OER electrocatalysts and analyze the effects of their specific surface area, oxidation stability, and conductivity.

### 4.3 Oxygen Reduction Reaction

ORR plays a critical role in many fields, especially in energy conversion devices including fuel cells and metal–air batteries. For proton exchange membrane fuel cells (PEMFC), ORR is the rate‐limiting reaction, which is the key part for production of electricity and the most important factor to the performance of a fuel cell.^250^ Research into ORR has attracted considerable attention in the past decades, but it still suffers from the sluggish kinetics.^24^ The overall process can be clarified by a most commonly accepted four‐electron pathway as follows

(4.7) $$O_{2}+4H^{+}+4e^{-}\;\;\to \;\;H_{2}\mathrm{O(inacidmedia)}$$

(4.8) $$O_{2}+2H_{2}O+4e^{-}\;\;\to \;\;4{\mathrm{OH}}^{-}\;(\mathrm{in}\;\mathrm{alkaline}\;\mathrm{medium})$$

It is a consensus that the future of fuel cells is mainly dependent on the breakthrough of ORR catalyst with high onset potential, low overpotential, and high catalytic currents. Previous study of ORR catalysts for alkaline fuel cells is mainly focused on Pt and Pt‐based alloys, and high catalytic performance has been demonstrated, but they have issues of instability and deactivation by CO poisoning and crossover effect. Current efforts are being devoted to searching for nonprecious catalysts with high ORR performance. Of these candidates, TMCs and TMNs are entering into people's vision.^104^, ^183^, ^251^ Regmi et al.^252^ prepared a series of high surface area TMCs and applied for HER and ORR. Their investigations indicated that some TMCs not only were good ORR catalysts but also good catalyst supports. TMNs are proven not attractive ORR catalysts due to their instability in O_2_ atmosphere.

#### 4.3.1 Tungsten Carbides for ORR

Previously we discussed that tungsten carbide is a highly active catalyst for several electrochemical reactions. When combined with platinum, tungsten carbide exhibited very high activity for the ORR reaction. W_2_C microspheres showed significant enhancement of electrochemical activity with a Pt load of 7.5 wt%, due to a claimed synergistic effect between Pt and W_2_C.^253^ A possible assumption to this synergistic effect could be described as follows. Strong support from W_2_C with high surface area can reduce the load mass of Pt and improve the utilization of expensive Pt. W_2_C and Pt have similar band structure and their heterojunction can tune the band structure toward better catalytic activities and more resistance to the CO poisoning. This point was supported by Liu et al.,^254^ who dispersed 3 nm of Pt nanoparticles onto high surface area WC crystals. However, they also found that the WC surface was unstable at potentials over 0.8 V and W*_x_*O*_y_* was formed during the reactions, lead to the detachment of Pt clusters from the support. Zellner et al.^255^ also reported that W_2_C phase remained stable only at potentials lower than 0.4 V, which was not as high as enough for an ORR electrocatalysts. In contrast, the WC film is stable at the anode potential below 0.6 V, demonstrating the potential to be used as an ORR electrocatalyst.

#### 4.3.2 Molybdenum Carbides for ORR

It is commonly believed that the surface Mo *d*‐band broadening gives rise to the catalytic properties resembling noble metals, which has been proved by several investigations.^256^ Nonetheless because of a diversity of molybdenum carbide structures, the theoretical model and the catalytic mechanism are still under debate.

**Figure 13:**
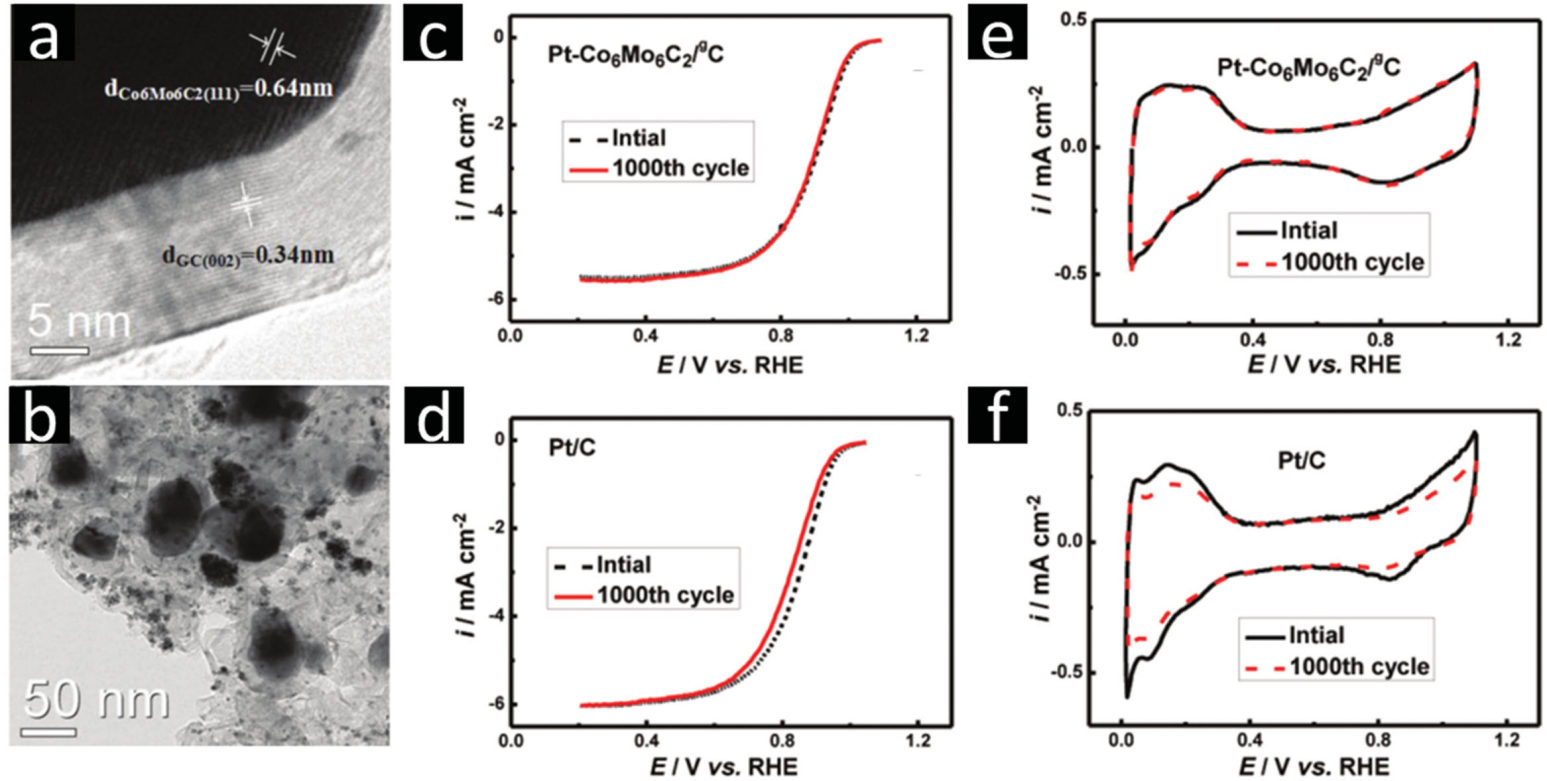
Cobalt molybdenum bimetallic carbide nanocomposites enhanced Pt catalyst for ORR. TEM images of a) graphitic carbon (*^g^*C) and b) Pt–Co_6_Mo_6_C_2_/*^g^*C. c,d) ORR curves of the two catalysts before and after 1000 cycles, and e,f) their corresponding CVs. Reproduced with permission.^189^ Copyright 2014, American Chemical Society.

A series of works have been devoted to molybdenum carbides composites with carbon materials such as CNTs and graphene. Pang et al.^257^ prepared nanosphere Mo_2_C particles with sizes of 3–6 nm which were well distributed on CNTs via a microwave assisted route, and the Pt‐Mo_2_C/CNTs electrodes with a Pt loading of 16 wt%. The samples exhibited higher surface area and ORR activity with a more positive onset potential in acid solution than those of Pt/CNTs under the same condition. In this electrode design, the formed CNTs network provides more active sites for the ORR reaction and also facilitates the transportation of electrons. Pt are well dispersed on the CNT network and thus can be utilized more effectively. In the meantime, the Mo_2_C may interact with Pt to form a kind of electronic ligand, which may lead to much better catalytic efficiency of Pt catalyst. But the authors had not conducted any further investigations on potential cycling stability. Ma et al. figured out that the strong negative electronic property of bimetallic carbides would change the surface electronic structure of Pt due to an electron‐donating effect, which might be the origin to the enhancement of ORR performance of the bimetallic carbide nanocomposites (Co_6_Mo_6_C_2_) supported Pt catalyst.^189^ Most importantly, the authors showed a clear evidence of the better stability of metal carbides composite catalyst in comparison with Pt/C after 1000 potential cycles (**Figure** **13**).

#### 4.3.3 Iron Carbides for ORR

Recently, a novel type of nonprecious metal catalysts based on iron carbide has been widely investigated toward ORR. Wen et al.^258^ synthesized nitrogen‐enriched Fe/Fe_3_C@C nanorods, which had higher kinetic current densities at different potential values than that of the Pt/C catalysts under the same testing conditions (e.g., 26.89 mA cm^−2^ for N‐Fe/Fe_3_C@C compared to 14.20 mA cm^−2^ for Pt/C catalysts at 0 V in neutral media). Lee et al.^259^ reported a simple two‐step approach based on impregnation and pyrolysis process to synthesize nanostructured Fe/Fe_3_C‐melamine foam composite, and showed the composite material possessed even better efficiency in transporting oxygen to the active sites for ORR than the Pt/C catalysts in alkaline media. Unfortunately, there was no information about the effect of the Fe_3_C phase to the catalysis property. Additionally, as the iron carbide tends to dissolve in acid, the possible usage of the Fe_3_C‐based catalysts in acidic electrolyte is worthy of further exploration.^260^ One effective approach is surface protection or wrapping. Chen et al.^261^ found that when iron nanoparticles were confined inside CNTs, catalytic functionalities could be observed on the outside of the CNTs due to a unique host–guest electronic interaction, which changed the local work function of the CNT walls. Their findings were also supported by Hu et al.,^262^ who developed an Fe_3_C/C catalyst in form of hollow spheres comprising Fe_3_C nanoparticles encaged inside graphitic layers. With the protection of graphitic layers, the Fe_3_C/C based catalyst exhibited an encouraging onset potential of 0.9 V in 0.1 m HClO, which was only about 0.1 V lower than that of Pt/C. Moreover, the catalyst showed excellent stability even in hot acid (0.5 m H_2_SO_4_ solution at 85 °C for 9 h) due to the protection of the surrounding graphitic layers. In this configuration, the inner Fe_3_C nanoparticles do not contact directly with the electrolyte, but activate the surrounding graphitic layers and make the outer surface of the carbon layer active in ORR. Such core–shell configuration with the unique synergetic effect may be used as a general strategy to develop active and durable ORR catalysts for fuel cell applications.

#### 4.3.4 Other Metal Carbides for ORR

As similar with the tungsten carbide and molybdenum carbide, other metal carbides such as titanium carbide and vanadium carbide also possess promising catalytic activity for ORR. Jalan et al. found that at 200 °C in phosphoric acid, Pt/TiC showed six times higher ORR activity than that of conventional Pt/C. Another group prepared TiC nanowires by a simple solvothermal method,^42^ which exhibited more positive onset potential than the bulk TiC particles and higher peak currents. Hu et al.^263^ reported that a synergistic effect between Pt and VC to account for the great improvement of catalytic activity for ORR. This improvement can be attributed to higher efficiency of Pt catalyst due to the interaction of VC as a kind of electronic ligand with the Pt. In addition to composite with Pt, bimetallic carbide nanocomposites for ORR reactions have also been explored. For example, Co_6_Mo_6_C_2_ nanoparticles (50 nm in size) uniformly dispersed on graphitic carbon was synthesized via an ion‐exchange method and applied as support for loading Pt nanoparticles. Such composite electrode showed superior ORR activity and exceptional stability compared with commercial Pt/C (see Figure 13) due to a synergistic effect.^189^ The bimetallic carbide might represent one type of promising materials for ORR due to more complex electronic configurations.

## 5 Conclusions, Challenges, and Prospects

Undoubtedly, energy storage and conversion technology and materials are the current research hotspot, and have therefore stimulated widespread interests in developing and refining more efficient electrode materials. One such device, fuel cell, when coupled with photoelectrochemical solar water splitting allows us to generate electricity effectively without pollution. Meanwhile, high‐performance EES devices (batteries and supercapacitors) are becoming major or backup power in electric vehicles and modern electronics. Whether fuel cell or EES devices, new and low‐cost active materials are the key to achieving performance enhancement and sustainability. In the past few years, there has been enormous research on high‐performance electrode materials that span from carbon, metal oxides, carbides, nitrides, and their derivatives. Nanotechnology has been playing a constructive role in improving the device performance.

TMCs and TMNs are emerging as new candidates as active materials (electrocatalysts) for applications in fuel cell and EES devices due to their high electrical conductivity, superior reactivity, and strong mechanical properties. Currently, various synthesis methods have been developed to fabricate TMCs and TMNs. For TMCs, either by template‐free or template‐assisted synthesis methods, they are all based on the carbothermal reactions. The precursor with metal sources need to be finally treated with carbon source at high temperatures to form TMCs. Template‐free synthesis methods have the disadvantage of poor control in morphology and size of TMCs. In contrast, the template‐assisted method allows much greater control and therefore the possibility of better understanding the effects of structure, morphology, and composition on the electrochemical properties. For TMNs, post ammonia reduction annealing is the main route to convert precursors into TMNs. Importantly, the core advantage of this conversion is that the morphology of the preformed precursors can be well preserved. Their conversion temperatures are usually lower than those of TMCs, but the usage of ammonia is not environmental friendly. Of course, the crystal structures and phases of TMCs and TMNs are also highly related with the annealing temperatures and duration. Furthermore, TMCs can serve as excellent backbone for the growth of other active materials forming multicomponent heterogeneous composites for fuel cell and EES applications.

In the light of high electrical conductivity and good reactivity, the TMCs and TMNs exhibit fascinating high‐rate capability for EES and superior catalytic reactivity for fuel cells. Nanostructured TMCs and TMNs are demonstrated generally with better performances (e.g., higher capacity and better electrocatalytic reactivity) than bulk counterparts. The enhancement performance is attributed to decreased particle size and higher surface area, which ensure sufficient contact between active materials and electrolytes or reactants, and provide short diffusion path for electrons and ions. Meanwhile, the excellent mechanical stability of TMCs and TMNs are important and favorable for the long‐term cycling life. The strong mechanical support can alleviate the volume change during reaction, and reduce the pulverization and keep the whole electrode stable. Hence, the TMCs and TMNs can also be suitable conductive supports/backbones for other active materials for EES application. Comparatively, the TMCs have superior chemical stability, while most TMNs are unstable in aqueous electrolyte, and even decompose during cycling. So the TMNs must be modified or coated by other protective layers to improve reactivity and cycling life. Additionally, because of high electrical conductivity, the TMCs and TMNs show better high‐rate capability, and lower anode potentials than the corresponding transition metal oxides when they are applied for EES. It is worth mentioning that nanostructured tungsten carbides and molybdenum carbides show impressive catalytic performance comparable to Pt‐based catalysts because of their similar energy band structures to Pt.

Apart from the obvious advantages of TMCs and TMNs, there are also some intrinsic drawbacks limiting their practical applications. First, challenge in large‐scale controllable synthesis. Fabrication of metal carbides and nitrides with controlled/specific structure and crystal planes usually requires multistep process and high temperature treatment, which make the process costly, complicated, or even uneconomical. This is especially serious in template synthesis. Furthermore, high crystalline quality and purity TMCs and TMNs are always formed at very high temperatures (TMCs > 1000 °C and TMNs > 800 °C). So new, low‐cost and green techniques are still desirable for preparation of high‐quality TMCs and TMNs. Second, instability in aqueous media. This is particularly problematic for TMNs, which nearly cannot be used as catalysts for fuel cells in aqueous media due to their instability in aqueous electrolyte. Doping of metal into TMNs is believed to be an effective solution to the problem of dissolution/decomposition. Third, difficulty in preferred orientation growth. The electrocatalytic reactions take place at the surfaces and interfaces between solids and gases or liquids, so the catalytic properties depend on the crystallographic planes. Evidence has been provided in MoS_2_ by Yi group, but systematic work is not reported yet for nitrides and carbides. Finally, the detailed electrochemical reactions of TMCs and TMNs are poorly understood as compared to metal oxides and carbon materials from the perspective of both thermodynamics and kinetics. More theoretical calculations and in situ proving techniques (such as in situ Raman spectroscopy, XPS, synchrotron X‐ray) could provide some insights.

Given the large family of TMCs and TMNs available in nanostructures (such as 2D layered structures) and their unique chemical/physical/electrochemical properties compared to metal oxides, there are still large room to explore. Mechanistic studies of the fundamental aspects of these materials in various energy applications are in urgent demand. In situ characterization techniques are needed for better understanding of exact formation/reaction processes including the way of lithium/sodium storage, the formation of Pt‐like behavior and the complex ion/electron transport behavior, and the reaction pathways. In particular, research on TMNs and TMCs in sodium ion batteries just commences and still needs further optimization of material structure and better understanding of the specific Na storage mechanism. Moreover, cost‐effective and highly reproducible synthesis methods are desirable to obtain TMNs or TMCs materials with tailored nanostructure and better performance. Finally, design and fabrication of advanced flexible and wearable TMCs/TMNs based electrodes for LIBs/SIBs is another important area to be explored urgently in pace of the advance in flexible electronics. This is a highly interdisciplinary topic that requires cooperation of researchers with complimentary expertise.
